# LncRNA *ZNF674-AS1* drives cell growth and inhibits cisplatin-induced pyroptosis via up-regulating CA9 in neuroblastoma

**DOI:** 10.1038/s41419-023-06394-8

**Published:** 2024-01-04

**Authors:** Kunming Zhao, Xinyi Wang, Yaqiong Jin, Xiaoxiao Zhu, Tao Zhou, Yongbo Yu, Xiaoying Ji, Yan Chang, Jiao Luo, Xin Ni, Yongli Guo, Dianke Yu

**Affiliations:** 1 School of Public Health, Qingdao University, 266071 Qingdao, Shandong Province China; 2 Beijing Key Laboratory for Pediatric Diseases of Otolaryngology, Head and Neck Surgery, MOE Key Laboratory of Major Diseases in Children, Beijing Pediatric Research Institute, Beijing Children’s Hospital, Capital Medical University, National Center for Children’s Health (NCCH), Beijing, China

**Keywords:** Long non-coding RNAs, Paediatric cancer, Cell death

## Abstract

Neuroblastoma (NB) is a challenging pediatric extracranial solid tumor characterized by a poor prognosis and resistance to chemotherapy. Identifying targets to enhance chemotherapy sensitivity in NB is of utmost importance. Increasing evidence implicates long noncoding RNAs (lncRNAs) play important roles in cancer, but their functional roles remain largely unexplored. Here, we analyzed our RNA sequencing data and identified the upregulated lncRNA *ZNF674-AS1* in chemotherapy non-responsive NB patients. Elevated *ZNF674-AS1* expression is associated with poor prognosis and high-risk NB. Importantly, targeting *ZNF674-AS1* expression in NB cells suppressed tumor growth in vivo. Further functional studies have revealed that *ZNF674-AS1* constrains cisplatin sensitivity by suppressing pyroptosis and promoting cell proliferation. Moreover, *ZNF674-AS1* primarily relies on CA9 to fulfill its functions on cisplatin resistance. High CA9 levels were associated with high-risk NB and predicted poor patient outcomes. Mechanistically, *ZNF674-AS1* directly interacted with the RNA binding protein IGF2BP3 to enhance the stability of *CA9* mRNA by binding with *CA9* transcript, leading to elevated CA9 expression. As a novel regulator of CA9, IGF2BP3 positively upregulated CA9 expression. Together, these results expand our understanding of the cancer-associated function of lncRNAs, highlighting the *ZNF674-AS1*/IGF2BP3/CA9 axis as a constituting regulatory mode in NB tumor growth and cisplatin resistance. These insights reveal the pivotal role of *ZNF674-AS1* inhibition in recovering cisplatin sensitivity, thus providing potential therapeutic targets for NB treatment.

## Introduction

Neuroblastoma (NB) is the most common extracranial solid tumor in children, accounting for approximately 12–15% of pediatric tumor-related mortality [1, 2]. NB commonly begins in one of the adrenal glands but can also develop in the chest, neck, abdomen, spine, or brain. The majority of NB cases (90%) are diagnosed in children under the age of 10, with an average age of approximately 18 months [3]. The exact cause of NB is still unclear, but it is believed to be associated with improper differentiation of neural crest cells into mature neurons of the sympathetic nervous system [4]. NB is highly heterogeneous and therapeutically challenging, where patients are assigned to low-risk, intermediate-risk, and high-risk subgroups on the basis of disease stage, age at diagnosis, and non-random chromosomal aberrations [5]. The majority of low-risk patients experience spontaneous regression, while those with high-risk subgroups have a survival rate of only 50% despite intensive therapy [6]. Therefore, there is a critical need to utilize biological markers to minimize inappropriate treatments and develop novel therapeutic strategies for NB patients in order to improve survival.

In cancer therapy, drug resistance remains a major challenge, leading to relapse and even mortality. Chemotherapeutic drugs primarily exert their antitumor effects by inhibiting cell proliferation and inducing regulated cell death (RCD), which limits tumor growth and causes tumor cell death [7, 8]. Recently, apart from apoptosis, other forms of RCD induced by chemotherapeutic drugs, such as pyroptosis, have been discovered [9, 10]. Pyroptosis is a newly identified form of RCD caused by pore-forming effector proteins called gasdermins, leading to cell swelling, the formation of large membrane bubbles, and perforation of the plasma membrane [11]. Recent studies have shown that chemotherapy drugs induce pyroptosis in tumor cells mediated by gasdermin E (GSDME), a key protein within the gasdermin family that is cleaved by activated caspase-3 [9, 12, 13]. In addition, the expression level of GSDME determines whether pyroptosis or apoptosis occurs in response to chemotherapy [9, 14]. Cells with high levels of GSDME undergo pyroptosis, whereas cells with low levels undergo apoptosis upon chemotherapy treatment. The expression of GSDME varies in different tissues and cell types. Interestingly, recent evidence demonstrates that the neuroblastoma SH-SY5Y cell line expresses high levels of GSDME. Furthermore, an analysis of the Cancer Genome Atlas (TCGA) cohort unveiled that neuroblastoma exhibits significantly higher levels of GSDME when compared with 31 other types of tumor tissues, second only to brain tumors [9, 15]. Considering the importance of GSDME activation in chemotherapy-induced tumor cell death, these findings raise the possibility that antitumor drugs like cisplatin (a first-line drug of NB chemotherapy) could induce pyroptosis in NB cells. However, the role of pyroptosis in the antitumor effect of cisplatin in NB, as well as the underlying regulatory mechanisms, remain poorly investigated.

In recent decades, mounting evidence has documented the vital roles played by the non-coding portion of the genome in many cancers. Long noncoding RNA (lncRNA) is a class of RNA transcripts with longer than 200 nucleotides without protein-coding potential [16]. It has been demonstrated that lncRNAs play central roles in a variety of fundamental biological processes and human diseases [17]. By interacting with proteins, lncRNAs regulate gene expression at various levels, including transcriptional, post-translational, and translational regulation, as well as protein activation or degradation [18]. In the context of NB, aberrant expression of certain lncRNAs has been shown to interfere with cell proliferation, cell death, migration, invasion, and even tumor initiation and progression [19–22]. Increasing evidence demonstrates that lncRNAs may also play important roles in determining NB chemotherapy sensitivities. For example, the depletion of lncRNA NBAT1 provides resistance to genotoxic drugs by limiting p53 accumulation in the nucleus and mitochondria by altering the function of CRM1 [23]. SNHG16 contributes to cisplatin resistance in NB by modulating the miR-338-3p/PLK4 pathway [24]. Furthermore, a growing body of studies reveals that lncRNAs are emerging regulators of cell pyroptosis [25–27]. Nonetheless, whether and how lncRNAs modulate chemotherapy drug-induced pyroptosis of NB and whether they resist the antitumor effects of chemotherapy drugs in NB have not been reported yet and require further investigation.

In this study, we identified an up-regulated lncRNA *ZNF674-AS1* in non-response patients to chemotherapy, and its high expression was correlated with poor prognosis. Previous studies have demonstrated that *ZNF674-AS1* is downregulated in non-small cell lung cancer and liver cancer [28, 29]. These studies have investigated the role of *ZNF674-AS1* in tumor inhibition, such as impeding cell proliferation and tumor metastasis. However, the specific role of *ZNF674-AS1* in regulating NB progression and its response to drug treatment has not been elucidated yet.

Here, we demonstrated that lower expression levels of *ZNF674-AS1* are associated with improved sensitivity to cisplatin-induced pyroptosis, a clinically relevant genotoxic drug. Additionally, *ZNF674-AS1* inhibition limited tumor growth both in vitro and in vivo. Moreover, mechanism studies revealed that *ZNF674-AS1* interacted with IGF2BP3 to regulate the transcriptional level of *Carbonic Anhydrase IX* (CA9), a key enzyme involved in tumor growth and correlated with the clinical prognosis of NB patients [30–32]. Importantly, the presence of *ZNF674-AS1* enhanced the association between IGF2BP3 and its target, *CA9* transcripts. This study, therefore, identifies *ZNF674-AS1* as a crucial target for optimizing genotoxic drug therapy in NB patients and targeting *ZNF674-AS1* may represent a potential treatment strategy for enhancing chemo-sensitization of anticancer drugs.

## Results

### LncRNA *ZNF674-AS1* is associated with a poor prognosis and chemotherapeutic resistance in neuroblastoma

**Fig. 1:**
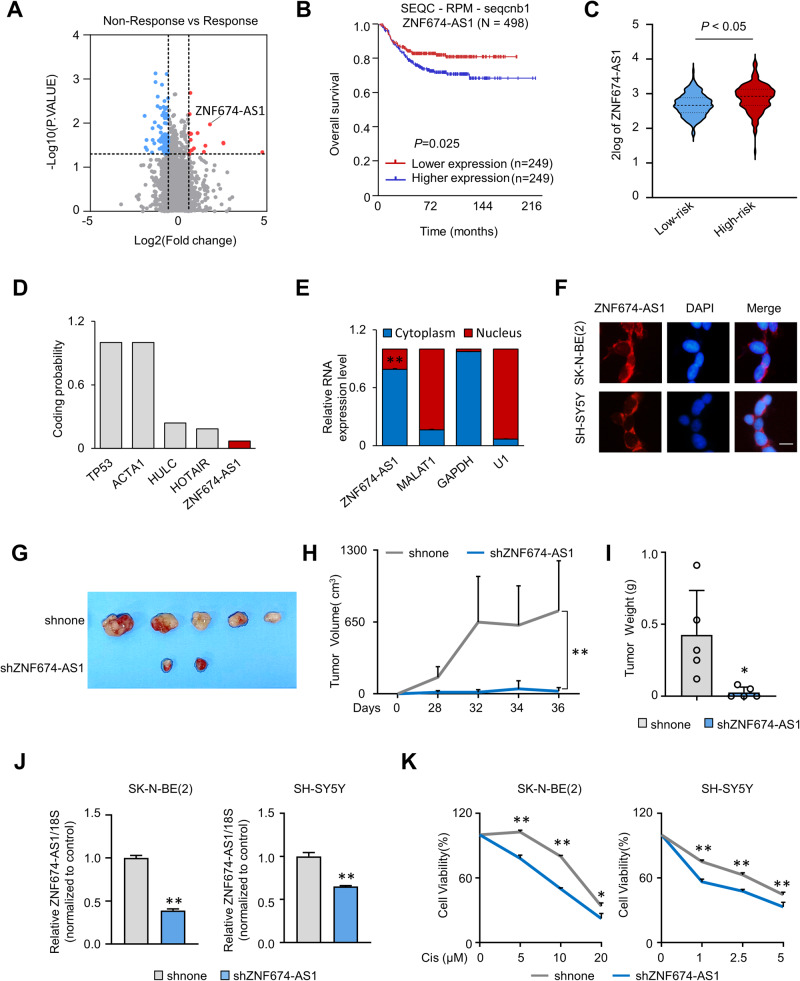
LncRNA *ZNF674-AS1* has a high expression associated with chemotherapeutic resistance and promotes neuroblastoma tumor growth. **A** Volcano plot revealed the differentially expressed lncRNAs between chemotherapy response and non-response neuroblastoma tissues. **B** Kaplan-Meier curve showed the overall survival of neuroblastoma patients according to the expression level of *ZNF674-AS1* in the SEQC-RPM-seqcnb1 dataset from the R2 platform (http://r2.amc.nl). **C** The expression levels of *ZNF674-AS1* in low- and high-risk neuroblastoma tissues. **D** The coding potential score of *TP53* and *ACTA1* (coding genes), *HULC* and *HOTAIR* (well-established long non-coding RNAs and *ZNF674-AS1* were identified by the online bioinformatic tool (Coding Potential Calculation, http://cpc.cbi.pku.edu.cn). **E** The expression of *ZNF674-AS1*, *MALAT1*, *GAPDH*, and *U1* in cytoplasm and nucleus were calculated by qRT-PCR after cell fraction separation. *MALAT1* and *U1* were used as nuclear markers, and *GAPDH* was a cytoplasmic marker. **F** The distribution of *ZNF674-AS1* in SK-N-BE(2) and SH-SY5Y cells was determined by RNA FISH assay. Scale bar, 50 μm. **G**–**I** Tumor volumes at the indicated dates (**G**), as well as images (**H**) and the tumor weights (**I**) for shnone and shZNF674-AS1 xenografts. The average values are presented as bar graphs (means ± SD) (n = 5 for each group). **J** The KD efficiency of *ZNF674-AS1* in SK-N-BE(2) and SH-SY5Y cell lines were calculated by qRT-PCR. **K** The relative cell survival rates of SK-N-BE(2) (left) and SH-SY5Y (right) stable *ZNF674-AS1* knockdown (KD) cells were measured after the indicated concentration cisplatin treatment. Data are derived from three independent experiments and presented as mean ± SD in the bar graphs. Values of controls were normalized to 1 (**J**, **K**). * *P* < 0.05; ** *P* < 0.01.

To identify key long noncoding RNAs associated with chemotherapeutic sensitivity in NB, we performed high-throughput RNA sequencing profiling of 35 NB patient tissues. Among these samples, 22 were nonresponsive to chemical therapy, while 13 showed a positive response. Differential expression analysis showed 100 differentially expressed lncRNAs, with 18 up-regulated and 82 downregulated lncRNAs (Fig. 1A). The top 10 upregulated candidates, including *ZNF674-AS1*, were identified (Fig. S1A). *ZNF674-AS1* stood out with the most significant *P*-value (Fig. 1A). Kaplan–Meier survival analysis of publicly available data from human NB tissues was conducted using the SEQC-RPM-seqcnb1 dataset from the R2 platform (http://r2.amc.nl) and revealed that high expression levels of *ZNF674-AS1* were associated with a poor prognosis in NB patients (Figs. 1B and S1B). In addition, *ZNF674-AS1* expression was found to be higher in high-risk neuroblastoma tumors (Fig. 1C). The results above indicate that *ZNF674-AS1* may play an important role in NB development and therapy.

Based on coding potential calculation, *ZNF674-AS1* showed a coding potential value of 0.069, slightly lower than well-characterized lncRNAs and significantly lower than protein-coding genes (Fig. 1D). Moreover, *ZNF674-AS1* was predominantly detected in the cytoplasm rather than the nucleus, by using cytoplasmic mRNA *GAPDH*, nuclear rRNA *U1* and nuclear lncRNA *MALAT1* as controls (Fig. 1E). This observation was further confirmed by fluorescence in situ hybridization (FISH) (Fig. 1F).

To understand the function of *ZNF674-AS1* in NB, we conducted xenograft experiments using stable knockdown (KD) *ZNF674-AS1* cells implanted into NSG mice. As shown in Fig. 1G–I, the reduction of *ZNF674-AS1* dramatically suppressed tumor growth. Furthermore, as expected, *ZNF674-AS1* KD significantly enhanced the susceptibility of NB cells to cisplatin-induced cell death (Fig. 1J, K). These findings strongly suggest that *ZNF674-AS1* promotes NB formation and progression and impedes the sensitivity of NB to chemical therapies.

### Cisplatin induces pyroptosis via caspase-3/GSDME in neuroblastoma cells

**Fig. 2:**
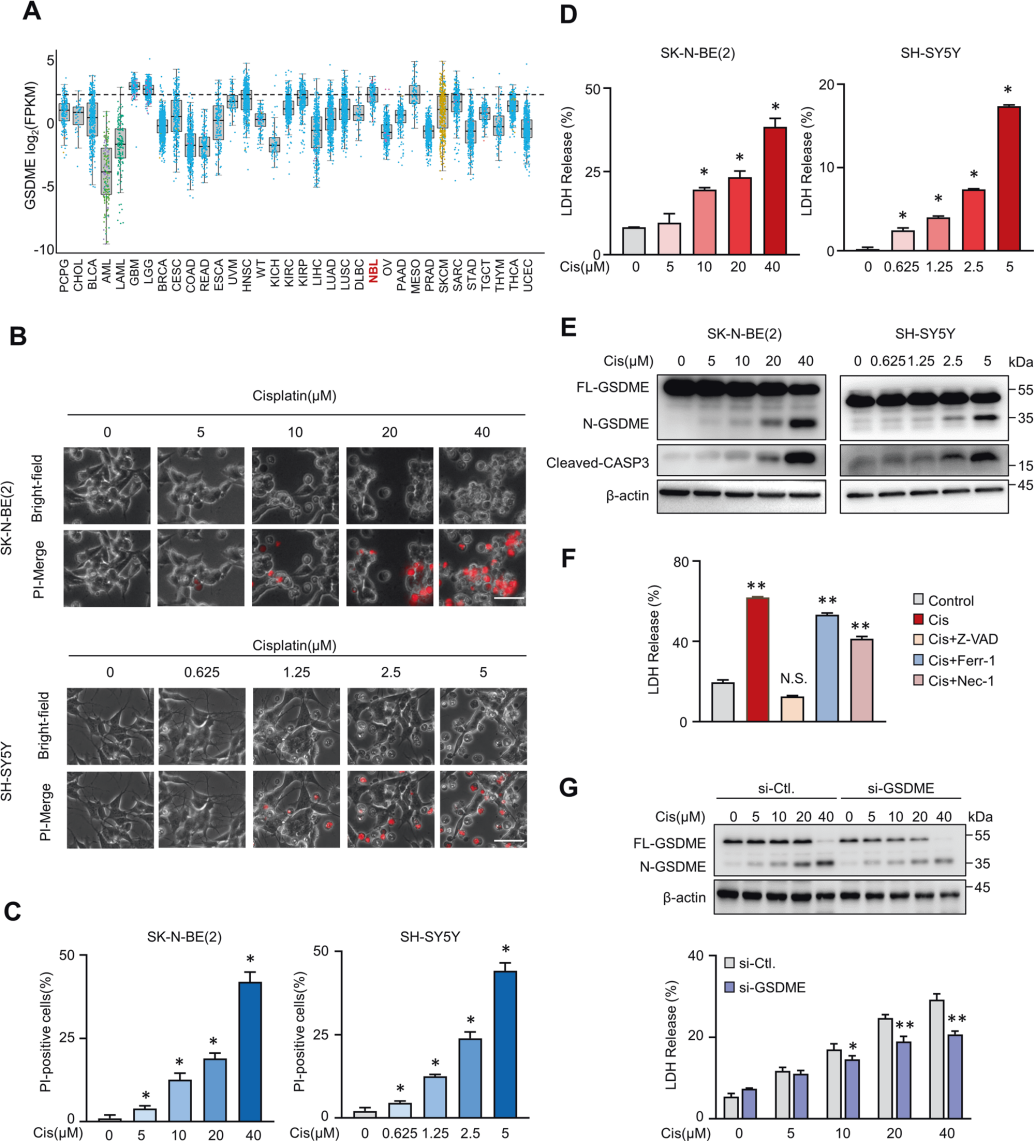
Cisplatin induces pyroptosis in neuroblastoma cells. **A** *GSDME* mRNA expression in various organ human tumor tissues in the TCGA cohort was applied from the TCGAportal database (www.tcgaportal.org). The center line in the box was the median, and NBL represented neuroblastoma. **B**–**D** Representative phase contrast images (**B**), PI-positive cells quantification (**C**), and LDH releasement (**D**) of SK-N-BE(2) and SH-SY5Y cells treated with indicated concentration cisplatin were presented. Scale bar, 50 μm. **E** Full length, N-terminal GSDME, and cleavage caspase-3 protein levels were detected with indicated concentration cisplatin treatment. **F** LDH releasement rates were analyzed after being treated with cisplatin with or without Z-VAD, Ferrostatin-1, and Necrostatin-1. **G** GSDME KD efficiencies and LDH release were determined in control (si-NC) and GSDME KD (si-GSDME) cells after the indicated concentration cisplatin treatment. Data are derived from three independent experiments and presented as mean ± SD in the bar graphs. * *P* < 0.05; ** *P* < 0.01.

After treating cells with cisplatin, we observed the morphology of dead cells induced by cisplatin differed from apoptosis. In contrast to apoptosis, the majority of dead cells exhibited swelling with plasma membrane blowing large bubbles, which is the typical characterization of cell pyroptosis (Fig. S2A). Previous studies have reported that chemical drugs can induce neuroblastoma cell SH-SY5Y to develop cell pyroptosis, which is associated with high-level endogenous GSDME expression [9]. The GSDME level determines whether cell death occurs in the form of apoptosis or pyroptosis [14]. Based on these clues, we analyzed the expression level of *GSDME* in various human tumors from TCGAportal (www.tcgaportal.org). Intriguingly, the *GSDME* mRNA was highly expressed in brain tumor, NB, and pleura among 34 tumor types (Fig. 2A). However, the expression of GSDMD, another pyroptosis inducer, showed a completely different profile (Fig. S2B). We then examined whether NB cells could undergo pyroptosis with cisplatin treatment. To this end, three NB cell lines were incubated with gradient concentrations of cisplatin. Pyroptosis levels were assessed by PI staining, LDH release, and N-GSDME cleavage detection. As expected, cisplatin-induced dose-dependent pyroptosis in SK-N-BE(2), SH-SY5Y, and IMR-32 cell lines (Fig. 2B–E and Fig. S2C–F). As GSDME can be specifically cleaved by active caspase-3 into N-GSDME fragment, the levels of N-GSDME were found to be correlated with cisplatin-triggered caspase-3 activation (Figs. 2E and S2F). More importantly, only the caspase inhibitor Z-VAD completely recovered cisplatin-induced lytic cell death (Fig. 2F). In addition, GSDME KD significantly attenuated cisplatin-induced cell death compared with control groups (Fig. 2G). Taken together, these data collectively suggest that cisplatin induces pyroptotic cell death in NB cells through the cleavage of N-GSDME by active caspase-3.

### *ZNF674-AS1* modulation regulates cisplatin-induced pyroptosis

**Fig. 3:**
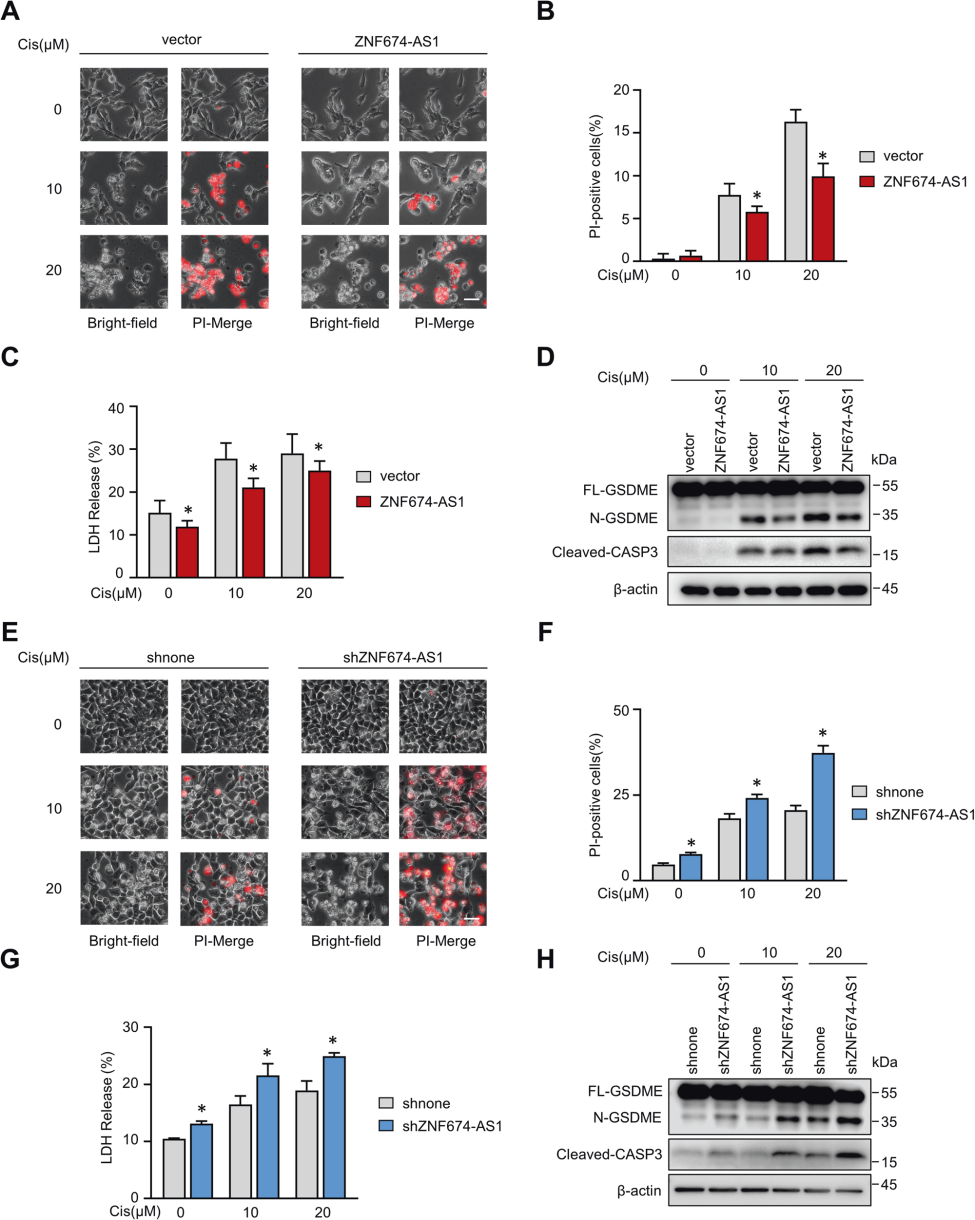
*ZNF674-AS1* represses cisplatin-induced cell pyroptosis. **A**–**C** Representative phase contrast images (**A**) PI positive statistic (**B**) and LDH releasement (**C**) of *ZNF674-AS1* overexpressed and control groups were showed after treated with 10 μM or 20 μM cisplatin. **D** Expression levels of full-length, N-terminal GSDME and cleavage caspase-3 protein of vector and *ZNF674-AS1* overexpressed cells were analyzed by western blot following indicated concentration cisplatin treatment. **E**–**G** Representative phase contrast images (**E**), PI positive statistic (**F**), and LDH releasement (**G**) of *ZNF674-AS1* KD and control groups were showed after treated with 10 μM or 20 μM cisplatin. **H** Expression levels of full-length, N-terminal GSDME and cleavage caspase-3 protein of control and *ZNF674-AS1* KD cells were analyzed by western blot following indicated concentration cisplatin treatment. Data are derived from three independent experiments and presented as mean ± SD in the bar graphs. Scale bar, 50 μm. * *P* < 0.05.

After observing the correlation between *ZNF674-AS1* and cisplatin sensitivity (Fig. 1K), we investigated whether *ZNF674-AS1* influences cisplatin-induced cell pyroptosis. Control and stable *ZNF674-AS1* overexpressed cells were treated with cisplatin in both SK-N-BE(2) (*MYCN* amplified) and SH-SY5Y (non-*MYCN* amplified) cells (Fig. S3A). Intriguingly, both bright-field and PI-merge images showed that *ZNF674-AS1* protected cells from cisplatin-induced cell death (Fig. 3A, B). In addition, the release of LDH, as well as N-GSDME levels, were markedly decreased upon *ZNF674-AS1* overexpression (Figs. 3C, D, and S3B). In contrast, endogenous *ZNF674-AS1* inhibition enhanced cisplatin-triggered pyroptosis (Figs. 3E–H and S3C, D). Collectively, these findings demonstrate that *ZNF674-AS1* inhibits cell pyroptosis induced by cisplatin, which is likely a common event independent of *MYCN*.

### *ZNF674-AS1* promotes neuroblastoma cell proliferation

**Fig. 4:**
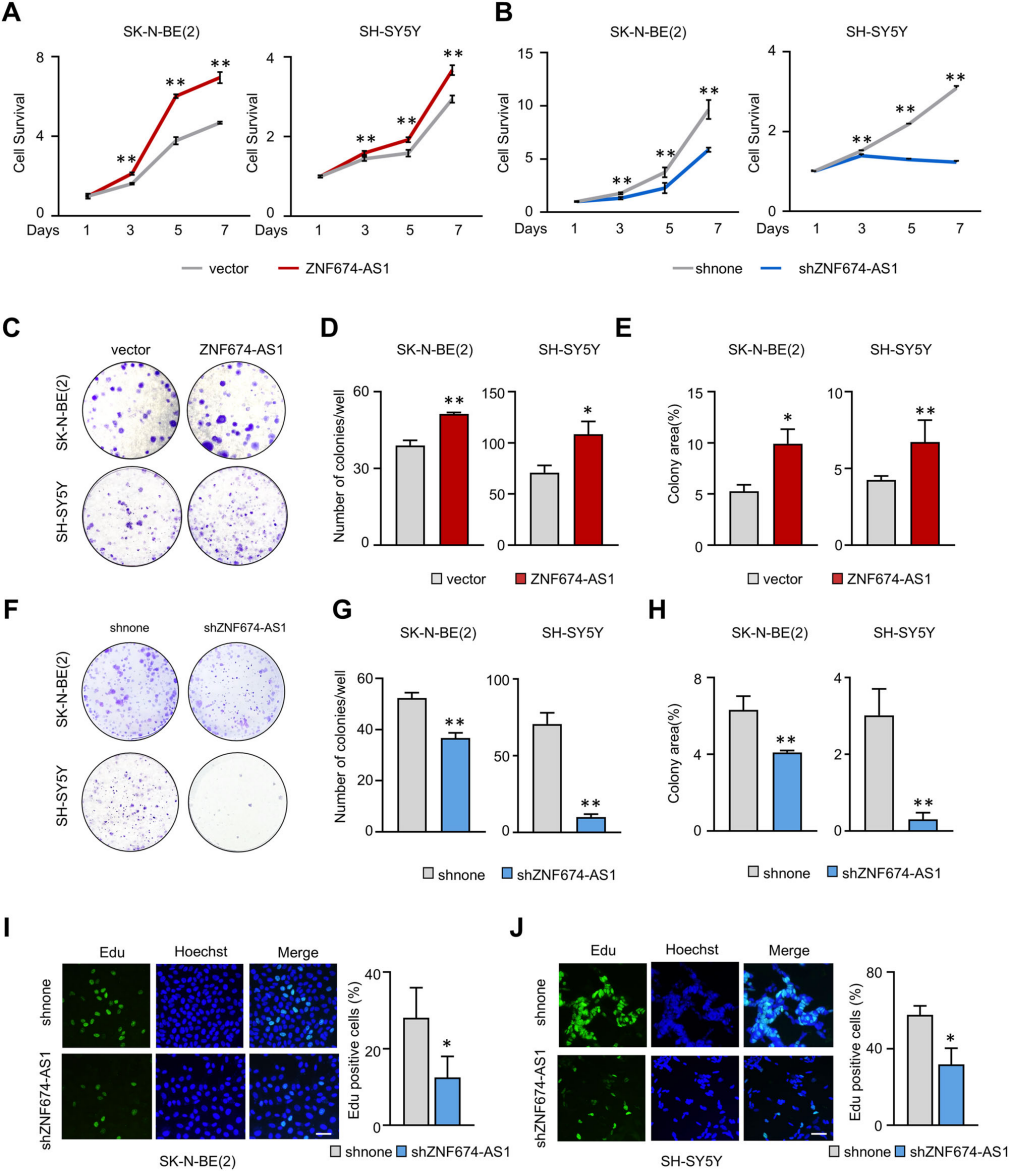
*ZNF674-AS1* promotes neuroblastoma cell proliferation. **A**, **B** The growth curves of *ZNF674-AS1*-overexpressing (**A**) and *ZNF674-AS1* KD (**B**) stable SK-N-BE(2) (left) and SH-SY5Y (right) cells were assessed by MTT assays. **C**–**E** Representative micrographs (**C**), colony numbers (**D**), and colony area (**E**) quantifications for *ZNF674-AS1*-overexpressing stable SK-N-BE(2) (upper) and SH-SY5Y (below) cells. **F**–**H** Representative micrographs (**F**), colony numbers (**G**), and colony area (**H**) quantifications for *ZNF674-AS1*-overexpressing stable SK-N-BE(2) (upper) and SH-SY5Y (below) cells. **I**, **J** Representative immunofluorescence images (left) and average Edu positive rates (right) of *ZNF674-AS1* KD SK-N-BE(2) (**I**) and SH-SY5Y (**J**) cells were presented by Edu incorporation assays. Scale bar, 50 μm. Data are derived from three independent experiments and presented as mean ± SD in the bar graphs. Values of controls were normalized to 1 (**A**, **B**). * *P* < 0.05; ** *P* < 0.01.

The tumor growth rate is a critical factor that determines responses to therapy and resistance. As a widely used and effective chemotherapeutic drug for NB, cisplatin is known to inhibit the proliferation and viability of NB cells [33]. Therefore, we next investigated the role of *ZNF674-AS1* in cell growth. As shown in Fig. 4A, ectopic overexpression of *ZNF674-AS1* in SK-N-BE(2) or SH-SY5Y cells led to a substantial increase in cell proliferation and colony formation (Fig. 4C–E). Conversely, *ZNF674-AS1* KD restrained clonogenic capacity and cell proliferation rate both in SK-N-BE(2) and SH-SY5Y cells (Fig. 4B, F–H, I, J). Consistent with the in vivo model (Fig. 1G–I), these data demonstrate the significant role of *ZNF674-AS1* in neuroblastoma cell proliferation and tumorigenesis.

### *ZNF674-AS1* acts through CA9 by interacting with IGF2BP3

**Fig. 5:**
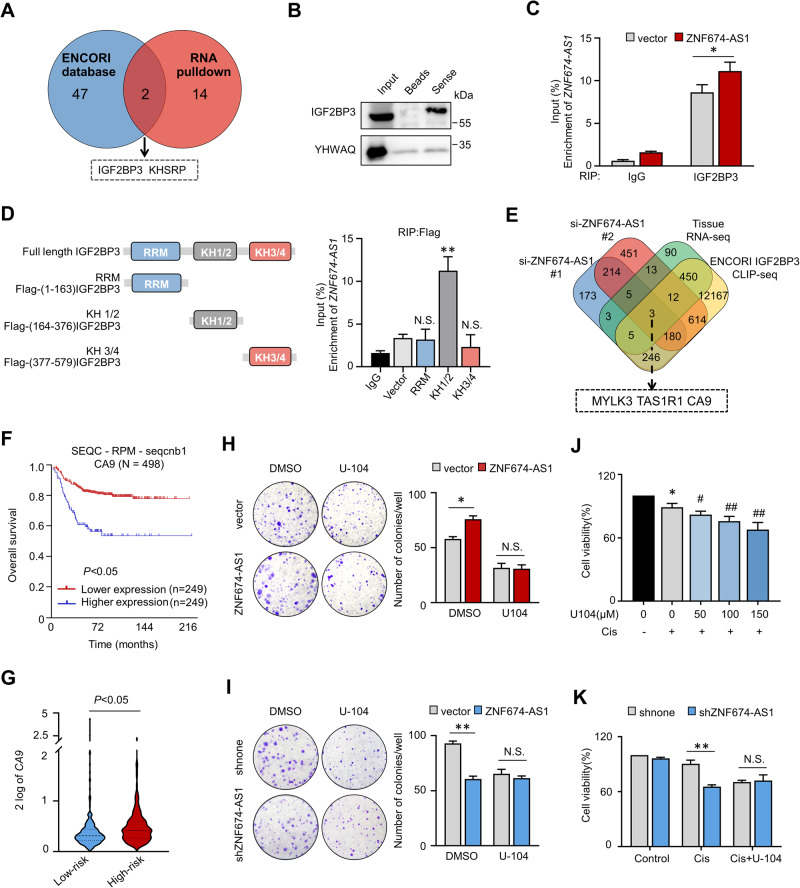
*ZNF674-AS1* plays oncogenic roles via CA9. **A** Venn diagram showing potential binding target genes by overlapping *ZNF674-AS1* RNA pull-down data and IGF2BP3 CLIP-sequence data. **B** IGF2BP3 protein levels in biotin-labeled *ZNF674-AS1* precipitates were analyzed by WB. YWHAQ was used as a negative control. **C** RIP assay was used to analyze the interaction between IGF2BP3 and *ZNF674-AS1* following IGF2BP3 immunoprecipitation in the vector and stable *ZNF674-AS1* overexpressing cells. IgG was used as a negative control. **D** *ZNF674-AS1* levels were detected by qRT-PCR following Flag RIP assay in SK-N-BE(2) cells after transfection of Flag-tagged truncations IGF2BP3, as indicated in the diagram shown left. **E** Venn diagram showing the potential target genes after integrating RNA-seq data of *ZNF674-AS1* KD by two independent siRNAs, RNA-seq data of neuroblastoma tissues, and IGF2BP3 CLIP-seq results. **F** The Kaplan-Meier curve showed the overall survival rate of neuroblastoma patients correlating with *CA9* mRNA expression. **G** The mRNA expression levels of *CA9* in low- and high-risk neuroblastoma tissues through the R2 platform. **H**, **I** The colonies image for control and *ZNF674-AS1* OE (**H**) /*ZNF674-AS1* KD (**I**) cells with or without U-104 treatment were presented (left), and the quantification of colonies numbers was shown in the bar graph (right). **J** The cell survival rates were detected after 10 μM cisplatin treatment cultured with indicating concentration U-104. **K** The cell survival rates of control and *ZNF674-AS1* KD cells were analyzed after cisplatin treatment with or without 100 μM U-104 for 24 h. Data are derived from three independent experiments and presented as mean ± SD in the bar graphs. Values of controls were normalized to 1 (**J**, **K**). * *P* < 0.05; ** *P* < 0.01; N.S., not significant.

The cellular localization of lncRNAs is closely associated with their molecular mechanisms. Cell fraction and FISH results demonstrated that *ZNF674-AS1* is mainly distributed in the cytoplasm (Fig. 1E, F), suggesting that *ZNF674-AS1* may exert its function by interacting with cytoplasmic protein. Therefore, a biotin RNA-protein pull-down assay followed by mass spectrometry analysis was applied to identify proteins that potentially interact with *ZNF674-AS1*. By comparing with the paired antisense control group, we selected 16 candidates which were reviewed in the UniProtKB database based on the number of unique peptides (more than one) obtained from the MS identification, as listed in Supplementary Fig. S4A. In addition, we profiled *ZNF674-AS1* interacting RBPs via CLIP-sequence data from the ENCORI database. Among these 49 RBPs, we focused on IGF2BP3 due to its predominantly cytoplasmic distribution rather than the nuclear-localized protein KHSRP (Figs. 5A and S4B). More importantly, the high expression of IGF2BP3 predicted poor prognosis of NB patients (Fig. S4C, D). The interaction between IGF2BP3 and *ZNF674-AS1* was validated by western blot. IGF2BP3 co-precipitated with biotin-labeled *ZNF674-AS1* in fresh cell lysate but not in the control beads group (Fig. 5B). In contrast, YWHAQ, which got the highest coverage score in the MS analysis result (Fig. S4A), did not bind to *ZNF674-AS1* (Fig. 5B). In addition, RNA immunoprecipitation (RIP) assay was applied to validate the physical interaction between *ZNF674-AS1* and IGF2BP3 in living cells. Compared with IgG, endogenous *ZNF674-AS1* exhibited significant interaction with IGF2BP3, and *ZNF674-AS1* overexpression led to increased binding with IGF2BP3 (Fig. 5C). We examined which domain in IGF2BP3 contributes to the interaction with ZNF674-AS1. Different IGF2BP3 truncations were generated, as indicated in Fig. 5D. Our data revealed that KH1/2 bound with *ZNF674-AS1* efficiently (Fig. 5D). Supportively, we found that IGF2BP3 inhibition improved caspase-3 activation and N-GSDME cleavage, and also completely abolished the impact of *ZNF674-AS1* (Fig. S5A). These data suggest that *ZNF674-AS1* fulfills its functions by interacting with IGF2BP3.

Next, to investigate the target genes that could be regulated by both *ZNF674-AS1* and IGF2BP3, we performed RNA sequencing by using two independent siRNA targeting *ZNF674-AS1*. Among the transcripts, a total of 224 genes were downregulated in both two *ZNF674-AS1* KD groups compared with the control group (Fig. 5E). Furthermore, by combining the *ZNF674-AS1* correlation genes in NB tissues and IGF2BP3 target genes obtained from CLIP-seq in the ECORI database, *MYLK3*, *TAS1R1* and *CA9* were identified as three potential candidate genes (Fig. 5E). *CA9*, but not *MYLK3* and *TAS1R1*, was finally selected as its high expression level was strongly associated with unfavorable prognosis and high-risk in NB patients (Figs. 5F, G and S5B).

In light of this discovery, we investigated whether the protumor effect or cisplatin insensitivity induced by *ZNF674-AS1* depended on CA9. To this end, CA9 was inhibited by a specific targeting inhibitor, U-104 (SLC-0111), which has been used in clinical trials [31]. In line with a previous report, U-104 significantly inhibited NB cell growth and accelerated caspase3 activation, which triggered N-GSDME cleavage (Figs. 5H, I and S5C-F). Importantly, the effect of *ZNF674-AS1* on cell proliferation was completely abrogated upon CA9 inhibition (Fig. 5H, I). Furthermore, considering that U-104 has been reported to enhance the effectiveness of chemotherapeutic drugs in killing cancer cells [32, 34], we wondered whether CA9 inhibition could improve the cisplatin sensitivity of NB cells. As shown in Fig. 5J, cell viability was slightly reduced by low levels of cisplatin, whereas U-104 treatment improved the cell viability inhibitory effects and pyroptosis levels (Fig. S5E) in a cisplatin dose-dependent manner. Consistent with the previous report, these data indicate that CA9 may be a potential target for enhancing the efficiency of chemical therapies. Furthermore, the effects of *ZNF674-AS1* KD on cisplatin-induced cell death were completely abolished by U-104 (Fig. 5K). Collectively, these data suggest that *ZNF674-AS1* alters cell proliferation and cisplatin-induced pyroptosis, mainly depending on CA9.

### *ZNF674-AS1* cooperates IGF2BP3 to promote CA9 expression

**Fig. 6:**
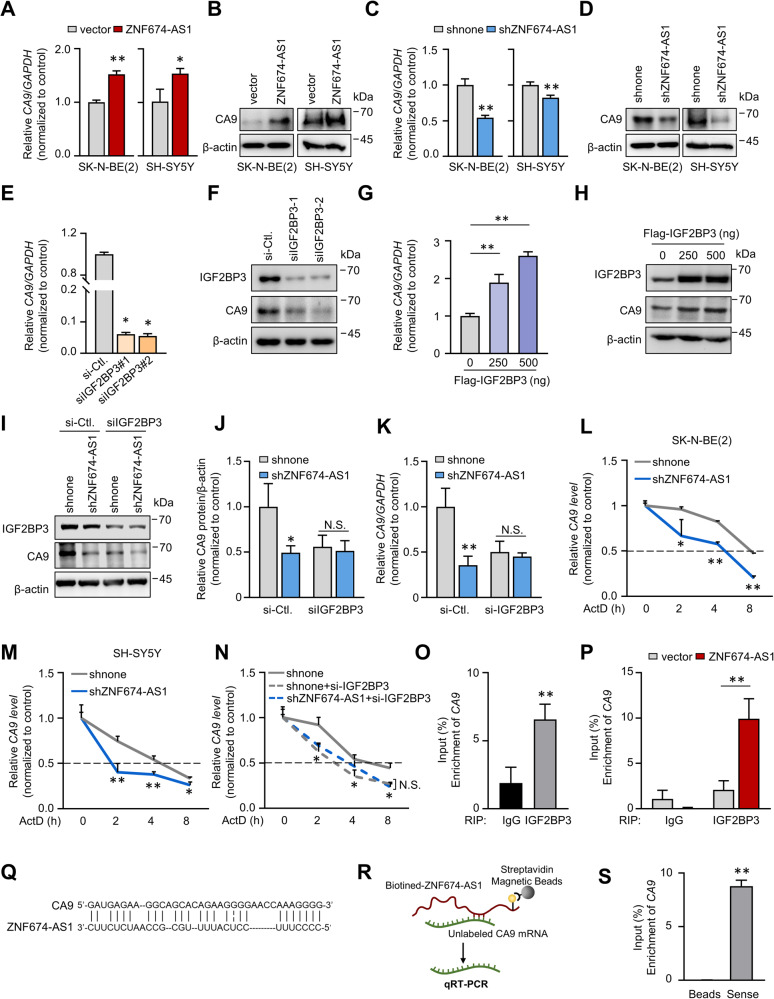
*ZNF674-AS1* elevates *CA9* mRNA stabilization through IGF2BP3. **A**–**D** The mRNA (**A**, **C**) and protein (**B**, **D**) levels of *CA9* were detected by qRT-PCR and WB in SK-N-BE(2) (left) and SH-SY5Y (right) stable *ZNF674-AS1* OE (**A**, **B**) or KD (**C**, **D**) cells. **E**, **F** The mRNA (**E**) and protein (**F**) levels of *CA9* were measured by qRT-PCR and WB after IGF2BP3 KD with two independent siRNA oligos. **G**, **H** *CA9* mRNA (**G**) and protein (**H**) expression levels were analyzed after indicating IGF2BP3 overexpressing plasmids transfected. **I**, **J** The mRNA (**I**) and protein (**J**) expression levels of *CA9* were measured by WB in *ZNF674-AS1* KD stable cells, with or without IGF2BP3 KD. **K**, **L** The half-lives of *CA9* mRNA in SK-N-BE(2) (**K**) and SH-SY5Y (**L**) *ZNF674-AS1* KD cells were measured by qRT-PCR in the presence of ActD. **M**, **N** qRT-PCR analyzed the CA9 mRNA half-lives in SK-N-BE(2) (**M**) and SH-SY5Y (**N**) *ZNF674-AS1* KD cell lines with or without IGF2BP3 KD. **O** *CA9* levels enriched by IGF2BP3 were detected by qRT-PCR following RIP assay. **P** qRT-PCR analyzed the enrichment of *CA9* by IGF2BP3 immunoprecipitating in vector and *ZNF674-AS1* OE cells. **Q** Schematic of predicted interaction between *ZNF674-AS1* and *CA9* according to IntaRNA (http://rna.informatik.uni-freiburg.de/IntaRNA/Input.jsp). **R** Schematic diagram of RNA-RNA binding assay. **S** *CA9* mRNA coprecipitate by biotin-ZNF674-AS1 determined by qRT-PCR after an RNA-RNA interaction assay. Data are derived from three independent experiments and presented as mean ± SD in the bar graphs. Values of controls were normalized to 1 **(A**, **C**, **E**, **G**, **J**, **M**–**P)**. * *P* < 0.05; ** *P* < 0.01.

To explore how CA9 is involved in *ZNF674-AS1* function, we examined the impact of *ZNF674-AS1* on *CA9* mRNA and protein levels. Intriguingly, *ZNF674-AS1* overexpression increased both *CA9* mRNA and protein expressions, while *ZNF674-AS1* KD inhibited their levels (Fig. 6A–D). Recent evidence has shown that IGF2BP3 could modulate RNA stability and improve protein expression of target genes [35]. Since CA9 was identified as a potential binding target of IGF2BP3, we examined the effect of IGF2BP3 ectopic overexpression or KD on the mRNA and protein levels of *CA9*. As expected, IGF2BP3 overexpression resulted in an induction of *CA9* mRNA and protein, while IGF2BP3 KD decreased their levels (Fig. 6E–H). More importantly, IGF2BP3 KD abrogated the ability of *ZNF674-AS1* to affect CA9 expression (Fig. 6I–K). Given that IGF2BP3 is known to bind to mRNA transcripts and control their stability, we then investigated whether *ZNF674-AS1*/IGF2BP3 was involved in the stabilization of *CA9*. Transcription inhibitor actinomycin D (ActD) was used to assess the *CA9* stability. As data shown in Fig. 6L, M, *ZNF674-AS1* KD accelerated the decay of *CA9* mRNA. Similarly, IGF2BP3 KD also led to faster degradation of *CA9* mRNA and abrogated the effect of *ZNF674-AS1* on *CA9* mRNA half-life (Fig. 6N). Furthermore, we determined whether *ZNF674-AS1* affected the interaction between IGF2BP3 and *CA9*. The binding of IGF2BP3 and *CA9* mRNA was confirmed by RIP assay. Compared with IgG, *CA9* mRNA was significantly enriched in IGF2BP3 antibody immunoprecipitate (Fig. 6O). Moreover, ectopic *ZNF674-AS1* overexpression dramatically enhanced the association between IGF2BP3 and *CA9* (Fig. 6P), demonstrating that the interaction between IGF2BP3 and *CA9* was regulated by *ZNF674-AS1*. We further explored how *ZNF674-AS1* fulfills such an ability and identified whether *ZNF674-AS1* could interact with *CA9* mRNA through unbiased prediction of RNA-RNA interactions by using IntraRNA [36]. As Fig. 6Q shows, the *CA9* transcript was recognized as a target of *ZNF674-AS1*. Furthermore, the result of an RNA-RNA binding assay confirmed this in silico prediction (Fig. 6R, S) [37]. These data suggest that *ZNF674-AS1* binds with *CA9* mRNA bridges and strengthens the interaction of IGF2BP3 with *CA9* mRNA, which contributes to increased *CA9* mRNA stability.

### *ZNF674-AS1* is clinically correlated with CA9 in human neuroblastoma tissues

**Fig. 7:**
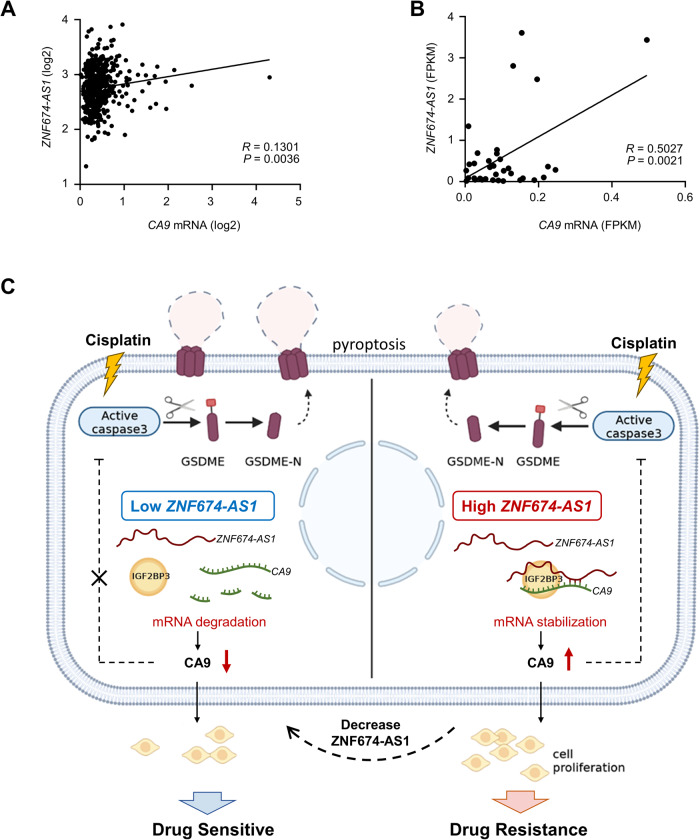
The association of *ZNF674-AS1* with *CA9* in human neuroblastoma tissues. **A** Correlation analysis of *ZNF674-AS1* with *CA9* mRNA in 498 human neuroblastoma tissues from the R2 platform. **B** Correlation analysis of *ZNF674-AS1* with *CA9* mRNA in 35 human neuroblastoma tissues. **C** Schematic diagram of the proposed model for *ZNF674-AS1*/IGF2BP3/CA9 axis modulating NB cisplatin sensitivity. Cytoplasmic *ZNF674-AS1* binds with *CA9* mRNA, recruits and elevates the capacity of IGF2BP3, and cooperates with IGF2BP3 to regulate their target transcript, *CA9*, leading to promote NB tumor development and limiting cisplatin-induced tumor cell pyroptosis. Such activity of *ZNF674-AS1* is restrained in NB cells due to si-*ZNF674-AS1* mediated *ZNF674-AS1* inhibition or *ZNF674-AS1* down-regulation, which recovers the sensitivity to cisplatin of NB cells. The diagram was created with BioRender.com.

In finding a novel *ZNF674-AS1*-CA9 axis, we assessed the clinical relevance between *ZNF674-AS1* and *CA9* in human neuroblastoma tissues. Intriguingly, *ZNF674-AS1* levels were positively correlated with increased *CA9* mRNA levels both in publicly available neuroblastoma tissues and in our own sequencing data (Fig. 7A, B).

Overall, our results demonstrate that lncRNA *ZNF674-AS1*, which significantly up-regulated in chemotherapy non-response NB patients, plays a crucial role in promoting NB tumor development and inhibiting cisplatin-induced tumor cell pyroptosis through up-regulating CA9 by binding IGF2BP3 (Fig. 7C). These findings suggest that targeting the *ZNF674-AS1*/CA9 axis may hold promise for developing more effective chemical therapeutic strategies.

## Discussion

The long-term survival rates of neuroblastoma patients, particularly those classified as high-risk, remain a significant concern. It is crucial to improve chemosensitivity and prevent or bypass chemoresistance to enhance the prognosis of NB patients [38]. Therefore, developing novel potential targets related to chemotherapeutic treatment response rates, as well as revealing the underlying mechanisms, is essential for optimizing clinical chemotherapeutical schemes and the treatment of NB. Emerging evidence has highlighted the involvement of lncRNAs, including NB, in tumor development [39]. By analyzing our RNA sequencing data, we identified lncRNA *ZNF674-AS1* as the most considerably up-regulated lncRNA in the chemotherapeutic non-response group compared to the chemotherapeutic response group. Remarkably, previous studies have suggested that *ZNF674-AS1* acts as a tumor suppressor and has been implicated in tumorigenesis in non-small-cell lung cancer [28]. However, the role of *ZNF674-AS1* in pediatric tumors, especially in neuroblastoma, remains unexplored. Analysis of publicly available databases revealed a strong correlation between high levels of *ZNF674-AS1* and poor patient outcomes, as well as the high-risk subgroup. Combining our sequencing data, these findings suggest that *ZNF674-AS1* may play a crucial role in the tumorigenesis of neuroblastoma and may contribute to the reduced efficacy of chemical therapies. It seems contradictory with the tumor suppressor function of *ZNF674-AS1* as described in published works. In fact, it is worth noting that functional molecules, including lncRNA, can exhibit discrepant functions in different cancer types [39]. For example, *NEAT1* drives prostate cancer progression through transcriptional regulation of prostate cancer-specific genes [40]. Intriguingly, *NEAT1* suppresses tumorigenesis in acute promyelocytic leukemia (APL) by accelerating APL cell differentiation [41]. These examples highlight the complexity of lncRNA functions and emphasize the need for a more comprehensive investigation into their underlying mechanisms, especially across different cancer types. These intriguing findings have encouraged us to explore the potential roles of ZNF674-AS1 in modulating NB progression further.

In this study, we found that *ZNF674-AS1* inhibition effectively limited NB tumorigenesis and sensitized NB cells to cisplatin-induced pyroptosis. The intracellular distribution of lncRNAs plays a decisive role in its molecular mechanism, lncRNAs can act as molecular decoys for RNA-binding proteins (RBPs), which cooperate with RBPs to regulate their targets [19]. Our data found that *ZNF674-AS1* predominantly localized within the cytoplasm of NB cells. Consistent with our findings, *ZNF674-AS1* has been reported to localize in the cytoplasm and regulate glycolysis and proliferation of granulosa cells by interacting with aldolase A (ALDOA) [42]. Our RNA pull-down assay and RIP assay provided evidence of the potential interaction between *ZNF674-AS1* and the RBP IGF2BP3. IGF2BP3 is an oncofetal protein related to pro-tumorigenesis in various cancer types by regulating target mRNA at the post-transcriptional level. Recent evidence suggests that IGF2BP3 may play crucial roles in regulating the proliferation and migration of NB cells [43, 44]. Additionally, high IGF2BP3 levels were related to NB patients’ poor prognosis (Fig. S4C, D). Consistently, our data suggest that *ZNF674-AS1* may play its regulatory role by recruiting IGF2BP3. Previous studies have reported the cooperative interactions between lncRNAs and IGF2BP3 in evaluating the mRNA stability of IGF2BP3 target genes [45]. In accordance with this idea, we identified Carbonic anhydrase IX (CA9) as the candidate target gene co-modulated by *ZNF674-AS1* and IGF2BP3. Importantly, we observed a positive correlation between the expression levels of both *ZNF674-AS1* and IGF2BP3 and the expressions of CA9. Furthermore, our findings provide evidence that *ZNF674-AS1* not only interacts with IGF2BP3 but also enhances its binding capacity to *CA9* by binding with the *CA9* transcript. However, the intricate molecular mechanism governing the interplay among these three molecular complexes needs to be further investigated. In addition, the upstream regulation mechanism of *ZNF674-AS1* is also important; whether *ZNF674-AS1* expression could be altered under different conditions, such as chemotherapeutic drug treatment, warrants further investigation. These will provide more information about chemotherapy insensitivity and create new opportunities for therapeutic intervention by repressing *ZNF674-AS1* in NB patients.

CA9 is a critical factor in tumorigenesis and has been identified as a potential therapeutic target for various cancers, including NB, due to its up-regulated expression in tumor tissues [46]. Additionally, increased CA9 expression in NB patients is inversely associated with overall survival and event-free survival, indicating its prognostic significance [30]. Several studies have demonstrated that CA9 up-regulation was associated with drug resistance, while the application of carbonic anhydrase inhibitors has shown promising effects in enhancing chemosensitivity [32, 46]. However, the association between CA9 and chemotherapy drug sensitivity in neuroblastoma has not been elucidated. Recently, Bayat et al. proposed that pre-inhibited carbonic anhydrases significantly potentiate the reduction of NB tumorigenesis by the HDAC inhibitor MS-275, indicating that CA9 inhibitor could be a promising therapeutic approach for NB patients [47]. Our study discovered that CA9 inhibition restrained cell proliferation and increased cisplatin-mediated pyroptosis in neuroblastoma cells by facilitating caspase3 activation. The connection between CA9 and caspase3 activation in NB cells remains to be investigated; whether CA9 may bind with other proteins to cooperate in modulating caspase3 activation warrants further investigation.

We identified lncRNA *ZNF674-AS1* as a novel epigenetic modulator of CA9 by prolonging its mRNA stability. The regulation of CA9 expression has been mainly studied at the transcriptional level, particularly involving the transcriptional factor hypoxia-induced factor (HIF-1), which recognizes the hypoxia response element (HRE) of *CA9* promoter and activates CA9 expression under hypoxia conditions [48]. Furthermore, various epigenetic regulators have been implicated in controlling CA9 expression. It has been reported that DNA methylation predominantly regulated CA9 expression in gastric cancer, while histone modifications [49], mediated by MORC2 and HDAC4, have been documented to control *CA9* transcriptional activation through histone H3 deacetylation [50]. However, the involvement of other epigenetic regulators, such as noncoding RNAs, in CA9 regulation remains poorly understood. Sabrina et al. demonstrated that miR-34a accelerated *CA9* mRNA degradation via base pairing with 3′UTR [51]. Notably, our findings provide additional regulators of CA9 that improve the stability of its mRNA. This discovery shed light on novel regulatory mechanisms that provide potential avenues for controlling CA9 expression.

Taken together, our findings highlight the crucial role of *ZNF674-AS1* in the regulation of cisplatin’s antitumor effects by inhibiting pyroptosis and promoting cell growth. Inhibition of *ZNF674-AS1* demonstrates a suppressive effect on NB initiation, indicating that targeting *ZNF674-AS1* could be a promising therapeutic strategy.

## Material and methods

### Cell culture and chemicals

Human neuroblastoma cell lines SK-N-BE2, SH-SY5Y, and IMR-32 were purchased from ATCC and cultured in Dulbecco’s modified Eagle’s medium (DMEM), MEM-F12, or MEM (contained 10 mM NEAA) supplemented with 10% (v/v) fetal bovine serum (Biological industries). All cells were maintained at 37°C in a humidified incubator (Thermo Scientific) with 5% CO_2_. Critical chemicals used in this study were shown as follows: Cisplatin (MedChemExpress, HY-17394), U-104 (MedChemExpress, HY-13513), and Actinomycin D (MedChemExpress, HY-17559). The cisplatin treatment concentrations of SK-N-BE(2), SH-SY5Y, and IMR-32 cells were based on their IC50s of cisplatin. The dosing time of cisplatin treatment was 24 h.

### Tissue samples

In our study, 35 cases of human neuroblastoma tissues were collected from the Beijing Children’s Hospital of Capital Medical University. All patients had undergone chemotherapy before surgery. The assessments of chemotherapeutic response were based on the 2015 neuroblastoma diagnosis and treatment expert consensus classification criteria. According to these classifications, 12 patients were classified as chemo-sensitive, while 23 patients were classified as chemo-resistant. The present study was approved by the Ethics Committees of Beijing Children’s Hospital. A total of 40 surgical specimens were immediately snap‑frozen in liquid nitrogen for subsequent total RNA extraction. Detailed clinical data are summarized in Table 1.

**Table 1:** Clinical characteristics of patients with neuroblastoma enrolled in this study (*n* = 35).

Parameter	Number of patients (%)
*Age at diagnosis (years)*	
<1.5	12 (34.29%)
1.5–3	10 (28.57%)
3–7	11 (31.43%)
>7	2 (5.71%)
*Sex*	
Male	19 (54.29%)
Female	16 (45.71%)
*MYCN status*	
Amplified	5 (14.29%)
Non-Amplified	25 (71.42%)
Not clear	5 (14.29%)
*Tumor stage (INSS stage)*	
I	0
II	1 (2.85%)
III	11 (31.43%)
IV	22 (62.86%)
IVS	1 (2.86%)
*MYCN, MYCN proto-oncogene*	

### Lentiviral production and stable cell establishment

The shRNA targeting *ZNF674-AS1* and the control sequences were 5′-CCGGGACTGGAATCCACCACTTACTCGAGTAAGTGGTGGATTCCAGTCTTTTTG-3′ and 5′-CCGGGCTGTGGCTCTAGACACTAAACTCGAGTTTAGTGTCTAGAGCCACAGCTTTTTG-3′) were synthesized by Tsingke Biotechnology (Beijing, China). The oligos were then subcloned into the PLKO.1 lentiviral vector. A full-length *ZNF674-AS1* transcript was synthesized and subcloned into a lentiviral expression vector, as previously reported [52]. Then, the lentivirus was packaged using the established protocol [53]. Briefly, both control and recombinant plasmids were co-transfected with two helper plasmids into 293 T cells. The lentivirus was collected 48 h after transfection and used to infect the target cells. Stable cells were selected with puromycin.

### Plasmids construction and siRNAs transfection

The Flag-IGF2BP3 plasmid was kindly provided by Prof. Sven Diederichs [54]. The indicated IGF2BP3 truncations were subcloned into a pcDNA3.1-3×Flag vector. The siRNA oligos used to target IGF2BP3 and GSDME were listed as follows: si-IGF2BP3-1(GGAATTGACGCTGTATAAT) and si-IGF2BP3-2(GAATCTTCAAGCACATTTA) for IGF2BP3; si-GSDME-1 (GCGGTCCTATTTGATGATGAA) and si-GSDME-2(GATGATGGAGTATCTGATCTT) for GSDME. All siRNAs were purchased from Tsingke Biotechnology (Beijing, China). These oligos were transfected into neuroblastoma cells by using Lipofectamine 2000 following the manufacturer’s instructions. After 48 h or 72 h of incubation, cells were collected for subsequent analysis.

### RNA extraction and real-time PCR

TriZol reagent (Takara) was used to extract total RNA by following the manufacturer’s protocol. mRNAs were reverse transcribed by The PrimeScript reverse transcription (TaKaRa, #RR047A) reagent kit with gDNA Eraser. Real-time PCR analysis was carried out by using QuantiNova™ SYBR Green PCR reagent (Qiagen, Duesseldorf, Germany) in the LightCycler® 480 System (Roche, Basel, Switzerland). Gene expression levels relative to 18 S snRNA or *GAPDH* were calculated using the 2^−ΔΔCT^ method. The primer sequences used in qRT-PCR are listed in Supplementary Table 1.

### Western blot assay

Total cell lysates were isolated using the urea buffer (8 M Urea, 1 M Thiourea, 0.5% CHAPS, 50 mM DTT, and 24 mM Spermine). Then, equal amounts of proteins (20 μg) were separated by SDS-PAGE and transferred onto a PVDF membrane. After incubating with indicated primary and secondary antibodies, the signals were detected using an enhanced chemiluminescence ECL kit (Boster, CA, USA). Antibodies used in the study were listed in Supplementary Table 2.

### RNA immunoprecipitation (RIP) assay

Cells were crosslinked with 1% formaldehyde for 10 min at room temperature; then, cells were harvested using lysis buffer supplemented with Protease Inhibitor Cocktail (MCE) and RNase inhibitor (Invitrogen). After sonication and centrifugation, the supernatants were collected and incubated with IgG or indicated antibodies overnight at 4°C. The complexes were then pulled down by Protein A/G after 2 h of incubation at room temperature. After five washes with wash buffer containing Protease Inhibitor Cocktail and RNase inhibitor, the immunoprecipitated RNAs were purified using Trizol and subjected to reverse transcription. The enrichment rate of the target RNA was analyzed by qRT-PCR.

### RNA pull-down assay and RNA–RNA binding assay

Biotin-labeled RNA was synthesized in vitro using the T7 promoter by biotin RNA labeling Mix (Roche, 11685597910). After removing DNA templates with RNase-free DNase I, the biotin-labeled RNA was subjected to secondary structure recovery for RNA pull-down assay. Then, it was incubated with fresh cell lysates for 1 h at 4 °C after being captured with streptavidin magnetic beads (MCE, HY-K0208). Beads were then collected and washed with a washing buffer 5 times. Finally, the bound proteins were denatured using an SDS loading buffer and then analyzed by mass spectrum or WB. For RNA-RNA binding assay, biotin-labeled ZNF674-AS1 was captured with streptavidin magnetic beads for 2 h at 4 °C, then incubated with *CA9* mRNA for overnight at 4 °C. After washing for 5 times, the amount of CA9 was detected by qRT-PCR.

### RNA fluorescence in situ hybridization (FISH) assay

The FISH assay for *ZNF674-AS1* was performed by using a lncRNA FISH kit obtained from Genepharma (Shanghai, China) following the manufacturer’s protocol. Briefly, cells adhered to the slides were fixed with 4% paraformaldehyde solution and then permeabilized using triton X-100. After blocking for 30 min at 37 °C, cells were incubated overnight at 37 °C with 1 μM Cy3-labeled probes in a hybridization buffer. After hybridization, cells were washed once with hybridization buffer and six times with 2× SSC buffer; the nuclei were then stained with DAPI for 5 min. Finally, images were acquired with a confocal microscope (Leica).

### Immunofluorescence staining

Immunofluorescence staining assay was carried out as our previous protocol described [55]. Briefly, after 4% paraformaldehyde fixing, 0.1% Triton X-100 permeabilizing, and 3% BSA blocking, cells were incubated with the antibody overnight at 4 °C. After that, cells were incubated with fluorescently labeled secondary antibody for 1 h and nucleus was stained by DAPI for 3 min. Images were captured by a confocal microscope (Leica).

### Cell proliferation and survival assays

Cell proliferation was measured using colorimetric MTT (MCE, HY-15924), colony formation, and Edu incorporation assays. For the MTT assay, control cells and stable *ZNF674-AS1* OE or KD SK-N-BE(2) and SH-SY5Y cells were seeded in 96-well plates. The cell proliferation rates at indicated time points were assessed by measuring the absorbances of formazans at 490 nm. Colony formation assay was assessed by seeding 1 × 10^3^ cells stable ZNF674-AS1 overexpression or KD SK-N-BE(2) and SH-SY5Y cells in 6-well plates. After culturing for 14 days, cells were stained using 0.5% crystal violet. The numbers of colonies (consisting of at least 50 cells) were counted by microscopy. Edu incorporation assays were performed following the manufacturer’s instructions. Briefly, stable *ZNF674-AS1* OE or KD cells were incubated with Edu (50 μM) for 2 h. After fixation with 4% formaldehyde, cells were stained with Apollo^®^ 488 and Hoechst 33342 to label Edu and the nucleus.

After drug treatment, cell survival was monitored using the CCK8 kit (MCE, HY-K0301) to indicate time. Cells were incubated with a 10% CCK8 solution for 2 h at 37 °C, and the absorbances were measured at 450 nm.

### Cellular fraction assay

The cytoplasmic and nuclear fractions of treated cells were isolated using the NE-PER Nuclear and Cytoplasmic Extraction Reagents kit (ThermoFisher Scientific) following the manufacturer’s protocol. Briefly, cells were lysed with cold CER I for 10 min, followed by the addition of CER II for a 1-min incubation on ice. After centrifugation, the supernatant was collected as the cytoplasmic component, while the pellet corresponded to the nuclear fraction.

### LDH release assay

The levels of LDH in the cell culture supernatants were analyzed using the LDH assay kit (Beyotime Institute of Biotechnology) according to the manufacturer’s protocol. Briefly, the supernatants were collected and incubated with LDH detection reagents in the dark for 30 min. After incubation, the absorbance was determined by a spectrophotometric microplate reader at 450 nm.

### PI staining assay

After treatment with the indicated concentration of cisplatin, cells were incubated with PI (10 μg/ml) in a cell incubator for 15 min. Then, the cells were photographed using an inverted fluorescence microscope (Olympus, Tokyo, Japan).

### RNA sequencing analysis

Total RNA was extracted from cells transfected with two independent siRNA targeting *ZNF674-AS1* and control cells and was subjected to high-throughput sequencing by Oebiotech (Shanghai, China). The strand-specific RNA libraries were constructed using TruSeq Stranded mRNA LTSample Prep Kit (Illumina, San Diego, CA, USA) according to the manufacturer’s instructions. Finally, these libraries were sequenced on the HiSeq2500 Illumina sequencing platform, and 125 bp paired-end reads.

### In vivo tumor xenografts

Tumor xenografts assay was conducted by following our previous protocol [56]. All animal procedures were performed according to the Rules for Animal Experiments published by the Chinese Government (Beijing, China) and approved by the Research Ethics Committee of Qingdao University, China. Briefly, 5 × 10^6^ control (shnone) or *ZNF674-AS1* KD (shZNF674-AS1) stable cells were subcutaneously inoculated into female M-NSG mice (4 and 5 weeks old). The tumor volumes were monitored every 3 days and calculated using the formula: length × width^2^ × 0.5. At the end of the experiment, mice were anesthetized and culled. M-NSG mice (Cat. NO. NM-NSG-001) were purchased from Shanghai Model Organisms Center, Inc.

### Statistical analysis

Statistical analysis was done using GraphPad software, version 8. Data are presented as means ± standard deviation (SD). A two-sided student *t*-test or One-way analysis of variance (ANOVA) was applied to assess the statistical significance. Correlations were calculated using Spearman or Pearson correlation coefficients. A *P* value less than 0.05 was considered statistically significant.

## Supplementary information

Supplementary file

Original westernblots

Reproducibility checklist

## Data Availability

All data are present in the manuscript and supplementary files. Additional data related to this paper may be requested from the corresponding author.
