# Correction: LncRNA *ZNF674-AS1* drives cell growth and inhibits cisplatin-induced pyroptosis via up-regulating CA9 in neuroblastoma

**DOI:** 10.1038/s41419-025-07371-z

**Published:** 2025-02-26

**Authors:** Kunming Zhao, Xinyi Wang, Yaqiong Jin, Xiaoxiao Zhu, Tao Zhou, Yongbo Yu, Xiaoying Ji, Yan Chang, Jiao Luo, Xin Ni, Yongli Guo, Dianke Yu

**Affiliations:** 1 School of Public Health, Qingdao University, 266071 Qingdao, Shandong Province China; 2 Beijing Key Laboratory for Pediatric Diseases of Otolaryngology, Head and Neck Surgery, MOE Key Laboratory of Major Diseases in Children, Beijing Pediatric Research Institute, Beijing Children’s Hospital, Capital Medical University, National Center for Children’s Health (NCCH), Beijing, China

**Keywords:** Long non-coding RNAs, Paediatric cancer, Cell death

Correction to: *Cell Death and Disease* 10.1038/s41419-023-06394-8, published online 04 January 2024

The original version of this article contained an error in Figure 4. We have carefully checked our original data and corrected Figure 4. We apologize for this error and state that this correction does not alter the interpretation of Figure 4 or the conclusions of the entire study.

**Original Figure 4** 
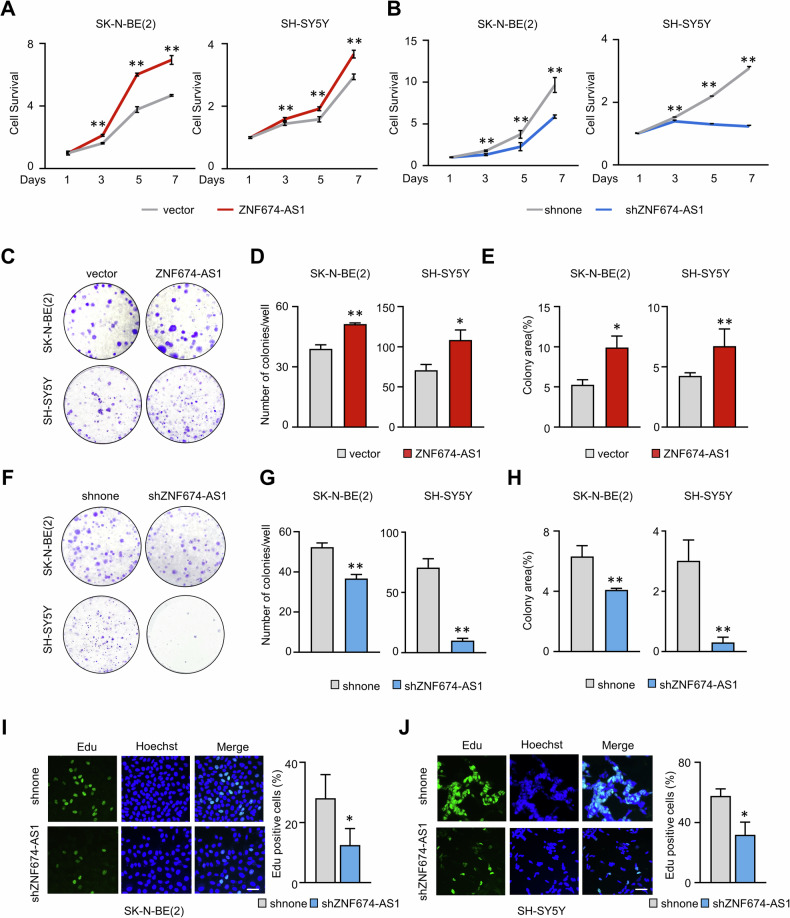


**Corrected Figure 4** 
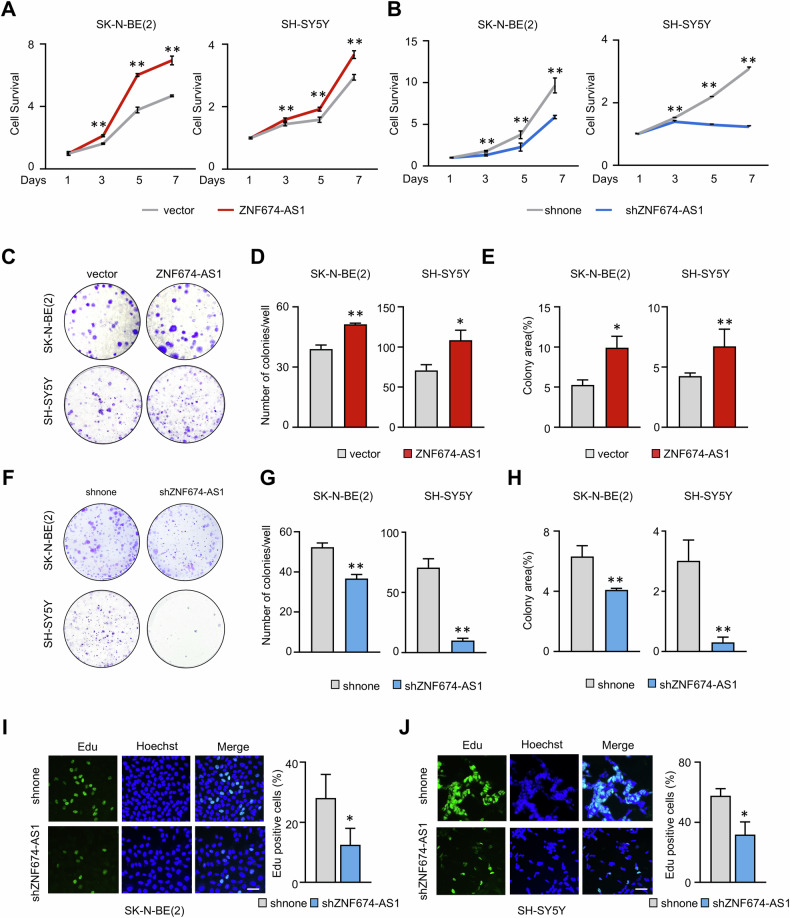


The original article has been corrected.

