# Supplementary material for: LncRNA ZNF674-AS1 drives cell growth and inhibits cisplatin-induced pyroptosis via up-regulating CA9 in neuroblastoma
Source: Cell Death Dis. 2024 Jan 4;15(1):5. doi: 10.1038/s41419-023-06394-8 (PMC10766958; doi:10.1038/s41419-023-06394-8)
Supplement: Supplementary file 1 — Supplementary file [file 41419_2023_6394_MOESM1_ESM.docx]

**Supplementary Figures**

**
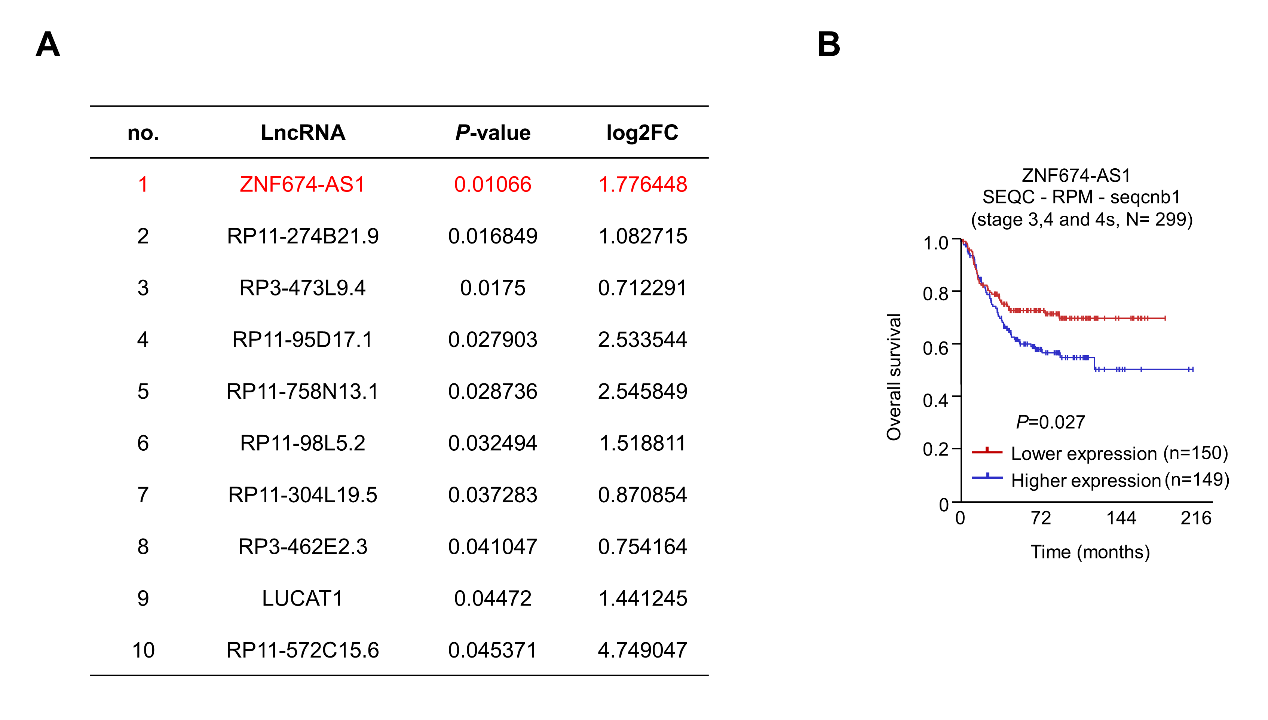
**

**Fig. S1 *ZNF674-AS1* is high expressed in chemotherapy non-response patients**

**(A)** The Table showed the top 10 up-regulated lncRNAs between the response and non-response patients after chemotherapy. **(B)** The Kaplan Meier curve showed overall survival rate of 299 stage 3, 4 and 4s neuroblastoma patients according to the levels of *ZNF674-AS1*.


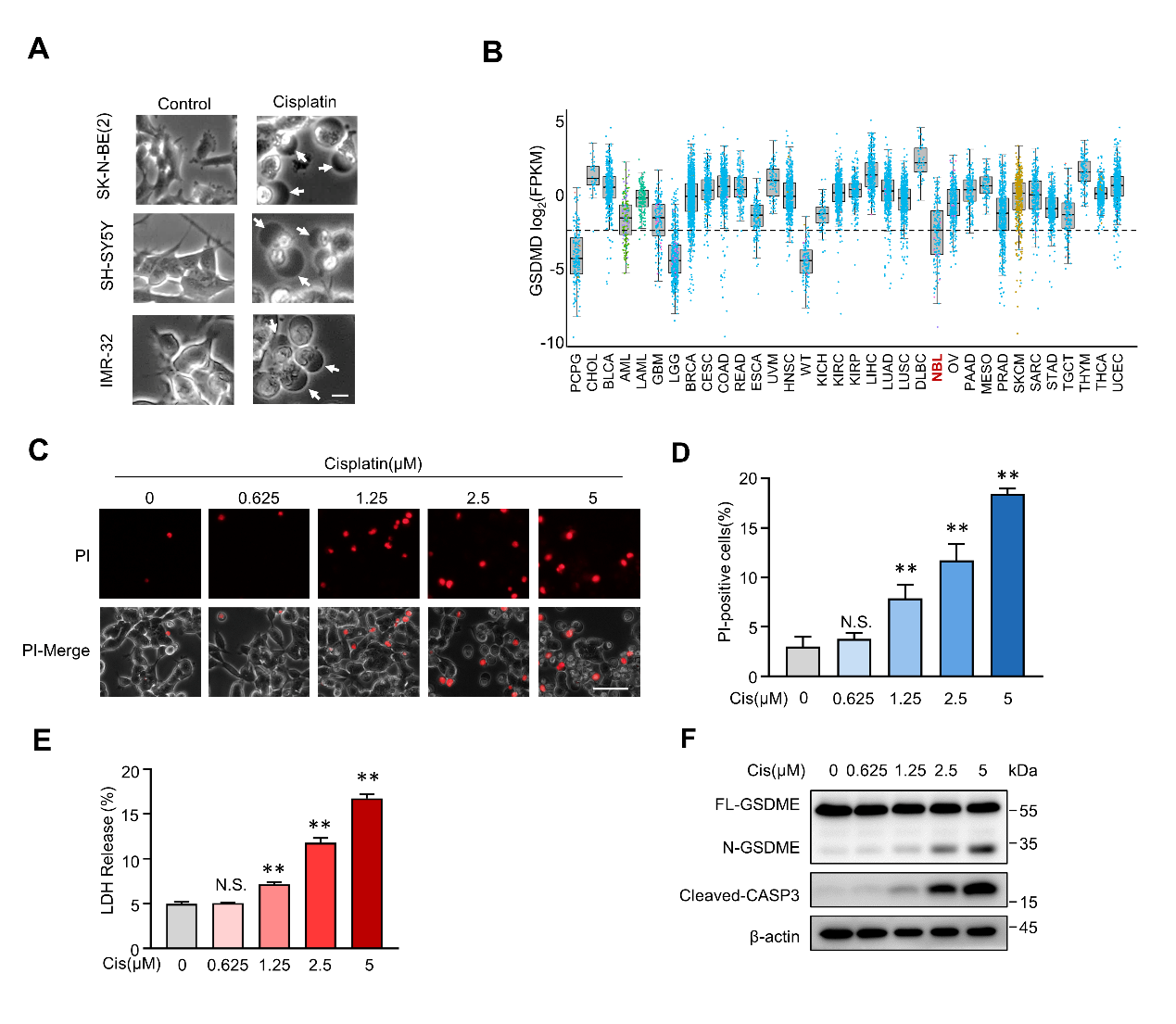


**Fig. S2 Cisplatin causes pyroptosis of neuroblastoma cells**

**(A)** Representative phase contract images of SK-N-BE(2), SH-SY5Y and IMR-32 cells with or without cisplatin treatment (arrows, the pyroptotic cells). Scale bar, 10μm. **(B**) *GSDMD* mRNA expression in various organ human tumor tissues in TCGA cohort were applied from TCGAportal database ([www.tcgaportal.org](http://www.tcgaportal.org)). The center line in the box was the median, and NBL represented neuroblastoma. **(C**-**F)** Representative phase contract images **(C)**, PI positive cells quantification **(D)**, LDH releasement **(E)** and expression levels of full length, N-terminal GSDME and cleavage caspase3 protein **(F)** of IMR-32 cells treated with indicated concentration cisplatin were presented. Scale bar, 50μm. Data are derived from three independent experiments and presented as mean ± SD in the bar graphs. ** *P* < 0.01; N.S., not significant.


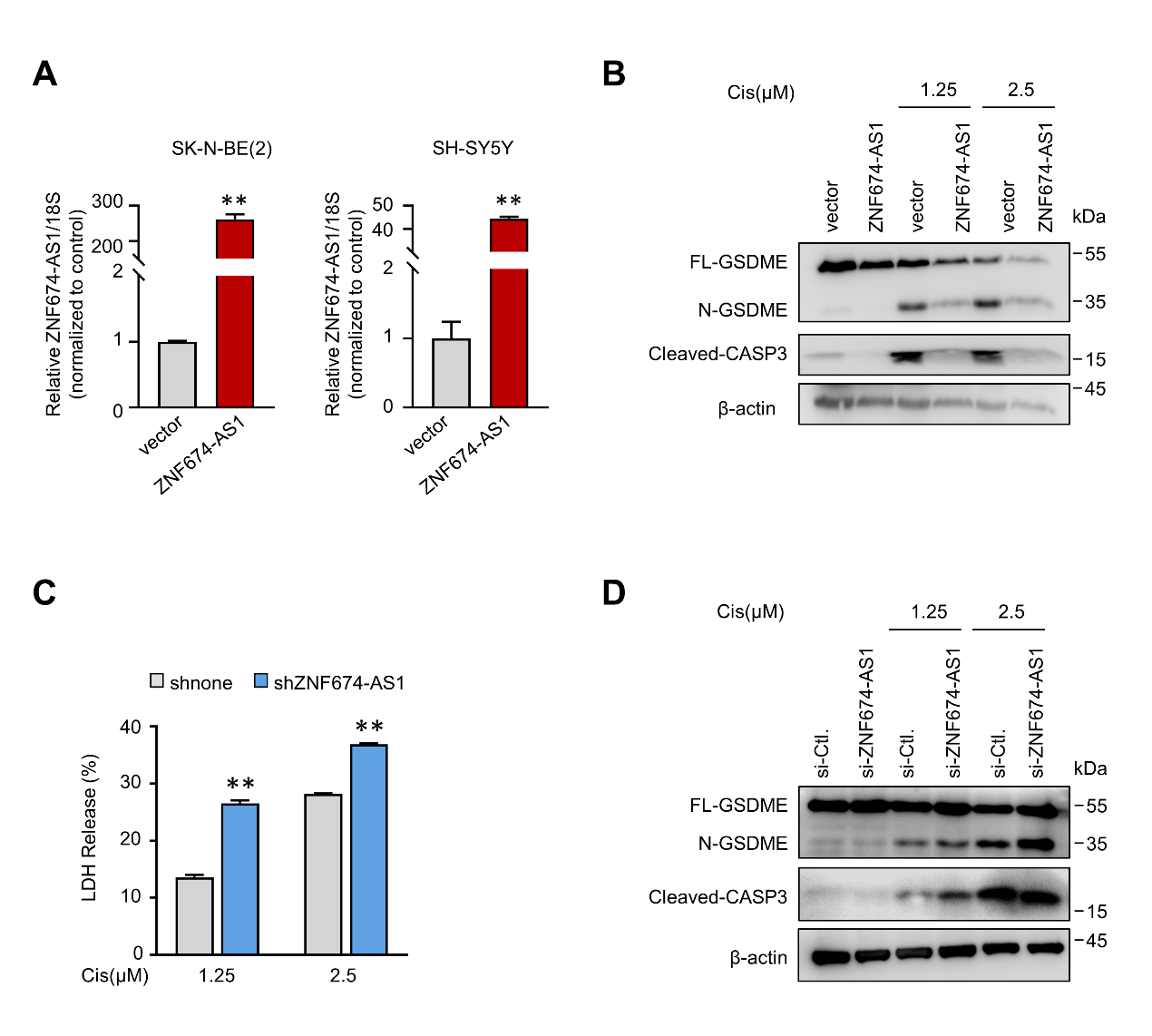


**Fig. S3 *ZNF674-AS1*** **suppresses cell pyroptosis triggered by cisplatin**

**(A)** The overexpression efficiency of *ZNF674-AS1* in stable SK-N-BE(2) and SH-SY5Y cell lines were calculated by qRT-PCR. **(B)** Expression levels of full length, N-terminal GSDME and cleavage caspase3 protein of vector and *ZNF674-AS1* overexpressed SH-SY5Y cells were analyzed by western blot following indicated concentration cisplatin treatment. **(C** and **D)** LDH releasement **(C)** and expression levels of full length, N-terminal GSDME and cleavage caspase3 protein **(D)** of control and *ZNF674-AS1* KD SH-SY5Y cells were analyzed by western blot following indicated concentration cisplatin treatment. Data are derived from three independent experiments and presented as mean ± SD in the bar graphs. Values of controls were normalized to 1 **(A** and **C)**. ** *P* < 0.01; N.S., not significant.


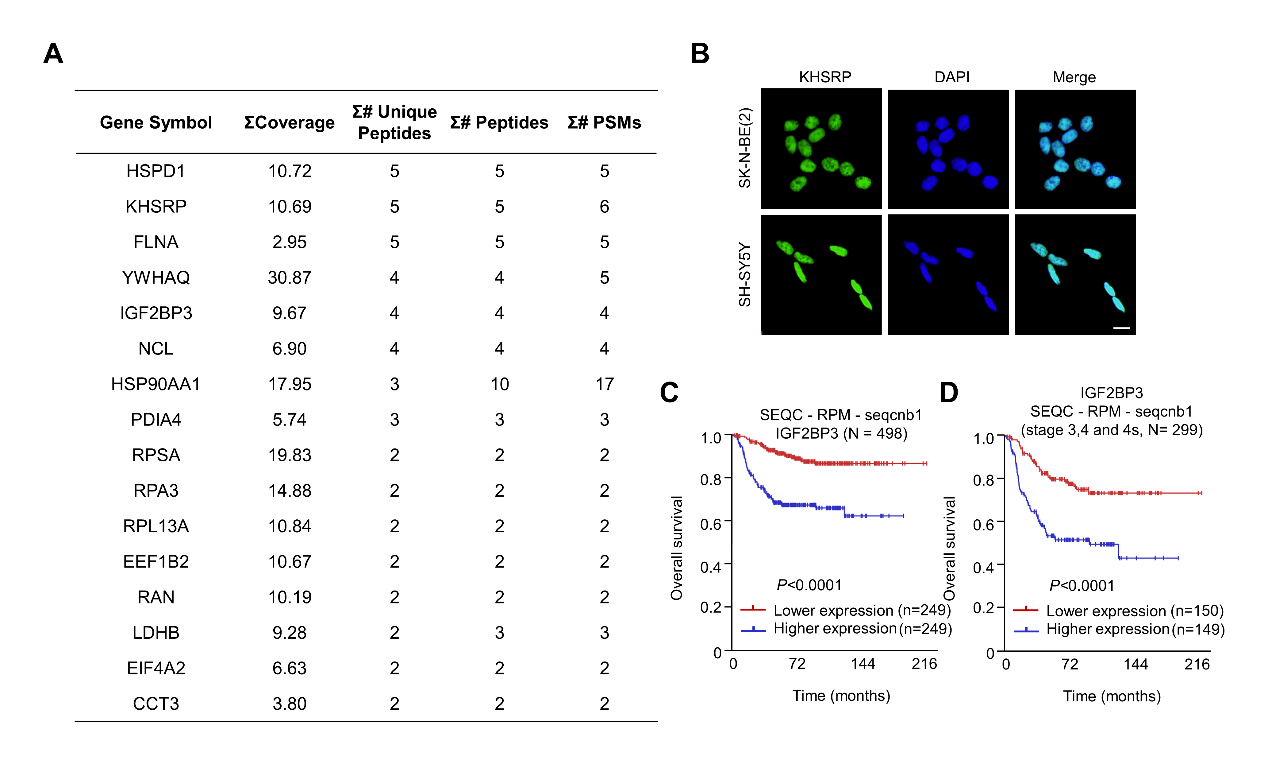


**Fig. S4 *ZNF674-AS1* bind with IGF2BP3**

**(A)** The table showed the candidate proteins interacted with ZNF674-AS1 detected by mass spectrometry analysis. **(B)** Cell distribution of KHSRP protein in SK-N-BE(2) and SH-SY5Y cells. Scale bar, 10μm. **(C)** Kaplan–Meier curves showed the probability of overall survival of neuroblastoma patients according to the levels of IGF2BP3. **(D)** The Kaplan Meier curve showed overall survival rate of 299 stage 3, 4 and 4s neuroblastoma patients according to the levels of IGF2BP3.


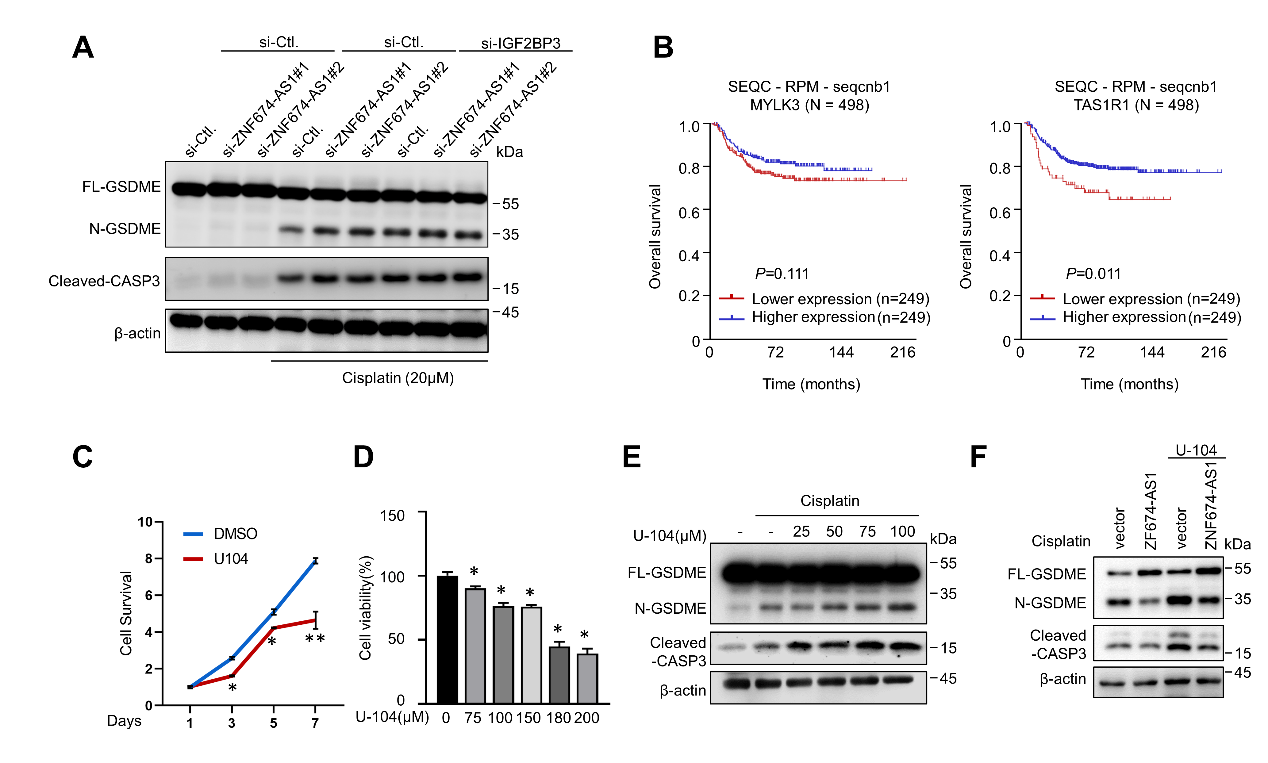


**Fig. S5 IGF2BP3 and CA9 impact neuroblastoma cell malignant phenotypes**

**(A)** Expression levels of full length, N-terminal GSDME and cleavage caspase3 protein were measured by WB after transfected with *ZNF674-AS1* targeting siRNAs, with or without IGF2BP3 KD, in the presence or absence of 20μM cisplatin. **(B)** The Kaplan Meier curve showed overall survival rate of neuroblastoma patients correlating with *MYLK3* (left) and *TAS1R1* (right) mRNA expression. **(C)** Cell proliferation rates were detected by CCK8 after U-104 treatment for indicating days. **(D)** Cell survival rates were measured by CCK8 with indicating concentration U-104 treatment. **(E)** Expression levels **o**f full length, N-terminal GSDME and cleavage caspase3 protein in SH-SY5Y cells were analyzed by WB following indicated concentration U-104 combinate with cisplatin (2.5μM) treatment. **(F)** Expression levels of full length, N-terminal GSDME and cleavage caspase3 protein of vector and ZNF674-AS1 overexpressed SK-N-BE(2) cells were analyzed by WB following cisplatin (20μM) and U-104 (100μM) combinate treatment. Data are derived from three independent experiments and presented as mean ± SD in the bar graphs. Values of controls were normalized to 1 **(C** and **D)**. * *P* < 0.05; ** *P* < 0.01; N.S., not significant.

**Supplemental Table 1 The list of Real-time PCR primer**

| **Real-time RT-PCR primer (5’-3’)** | | |
| --- | --- | --- |
| *ZNF674-AS1* | Tsingke Biotechnology | Forward: TCGCTCTAAGGGGAGAAGGAA  Reverse: TCCCAGGGTCTGGCTGTATT |
| *MALAT1* | Tsingke Biotechnology | Forward: GCGAGCTATGAACGCCTGG  Reverse: AGAGACCTTGTAATTGCGCG |
| *U1* | Tsingke Biotechnology | Forward: TGAGAGACAGCATCTCAAAGAC  Reverse: CCATCAGGTGCAGAAAGACAAG |
| *CA9* | Tsingke Biotechnology | Forward: GTGCCTATGAGCAGTTGCTGTC  Reverse: AAGTAGCGGCTGAAGTCAGAGG |
| *GAPDH* | Tsingke Biotechnology | Forward: GTCTCCTCTGACTTCAACAGCG  Reverse: ACCACCCTGTTGCTGTAGCCAA |
| *18S* | Tsingke Biotechnology | Forward: AACTTTCGATGGTAGTCGCCG |
|  |  | Reverse: CCTTGGATGTGGTAGCCGTTT |

**Supplemental Table 2 The list of antibodies**

| **Antibodies** | | |
| --- | --- | --- |
| Anti-GSDME | Abcam | Cat.#ab215191 |
| Anti-cleaved-caspase3 | Abcam | Cat.#ab32042 |
| Anti-IGF2BP3 | Proteintech | Cat.#14642-1-AP |
| Anti-YWHAQ | Santa Cruz | Cat.#sc-69720 |
| Anti-KHSRP | Zenbio | Cat.#R383225 |
| Anit-CA9 | Proteintech | Cat.#11443-1-AP |
| Anti-β-actin | BOSTER | Cat.#BM0627 |
| Rabbit IgG | Beyotime | Cat.#A7016 |
|  |  |  |
