# Supplementary figures and images for: LncRNA ZNF674-AS1 drives cell growth and inhibits cisplatin-induced pyroptosis via up-regulating CA9 in neuroblastoma
Source: Cell Death Dis. 2024 Jan 4;15(1):5. doi: 10.1038/s41419-023-06394-8 (PMC10766958; doi:10.1038/s41419-023-06394-8)

### Figure 2.

**E**

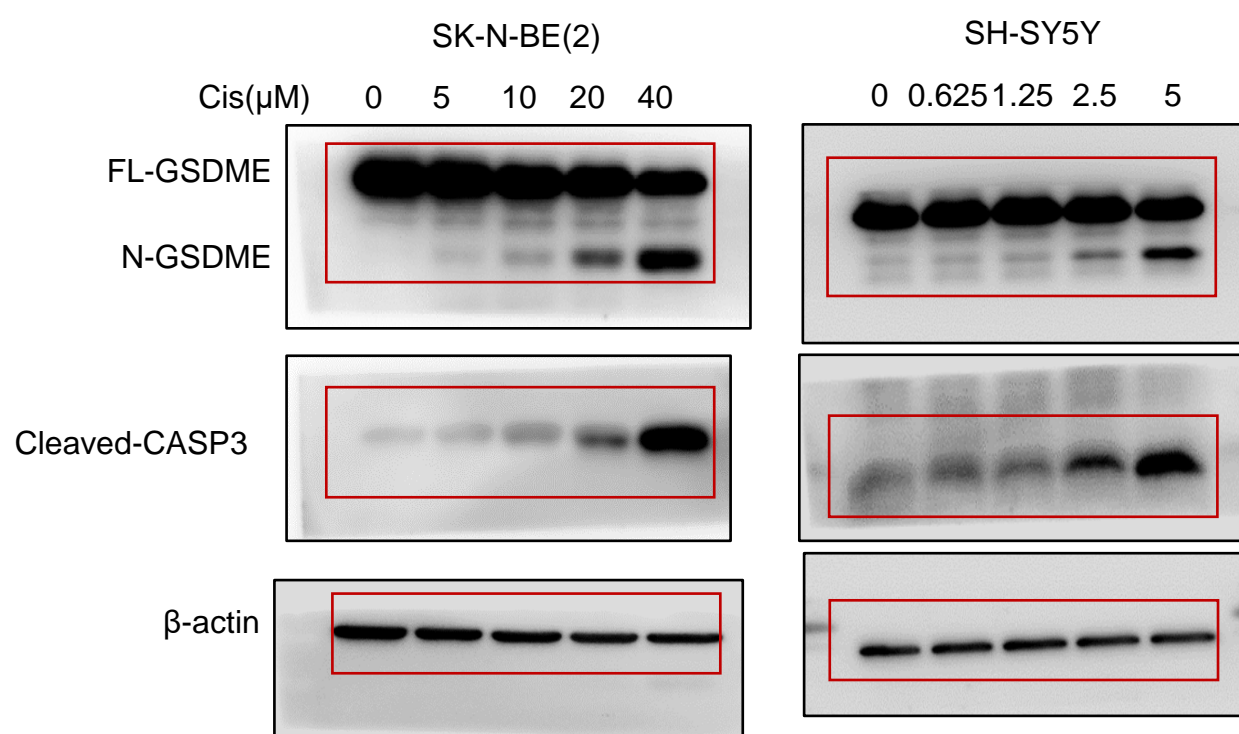

## G

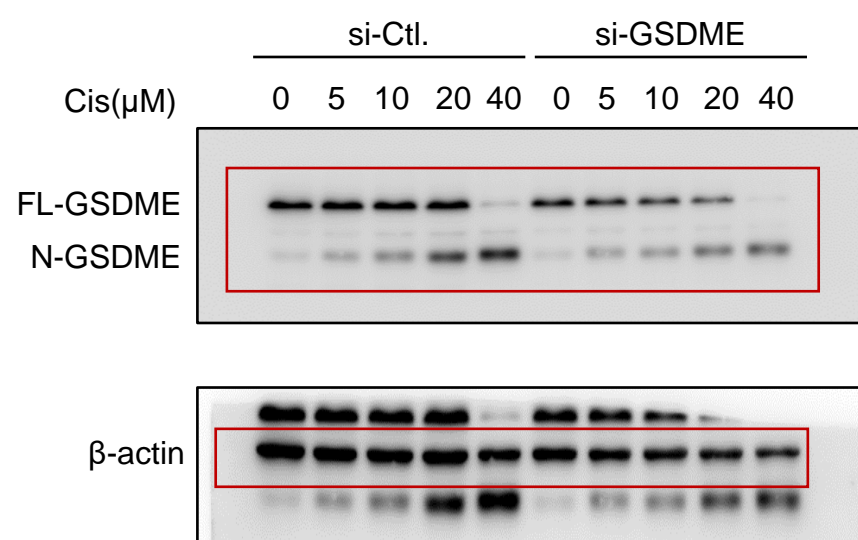

Figure 3.

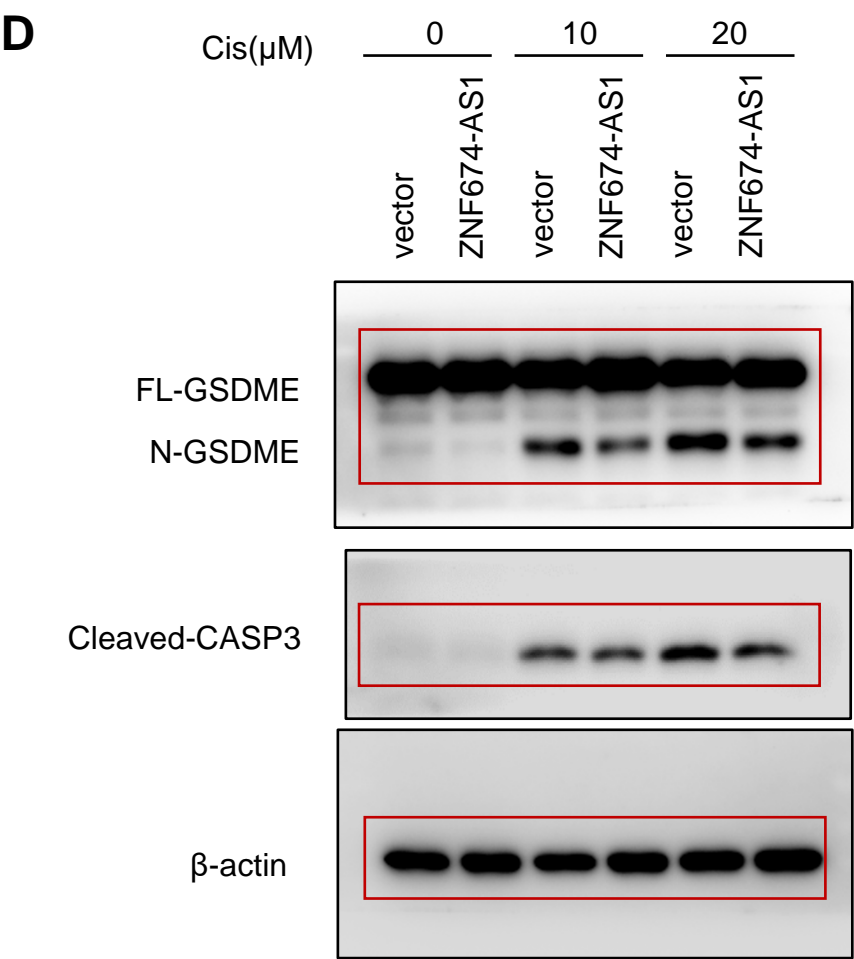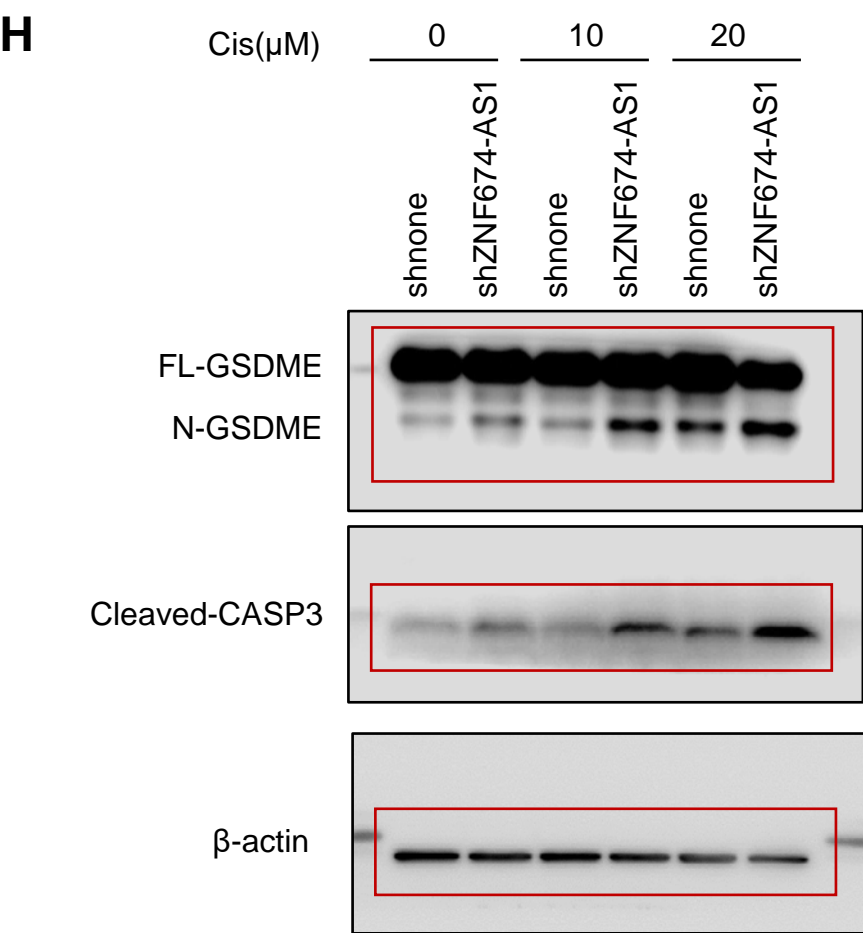

Figure 5.

B

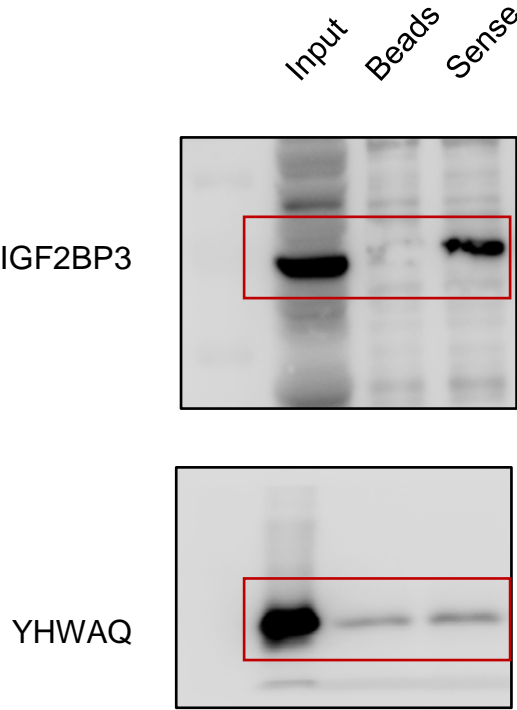

Figure 6.

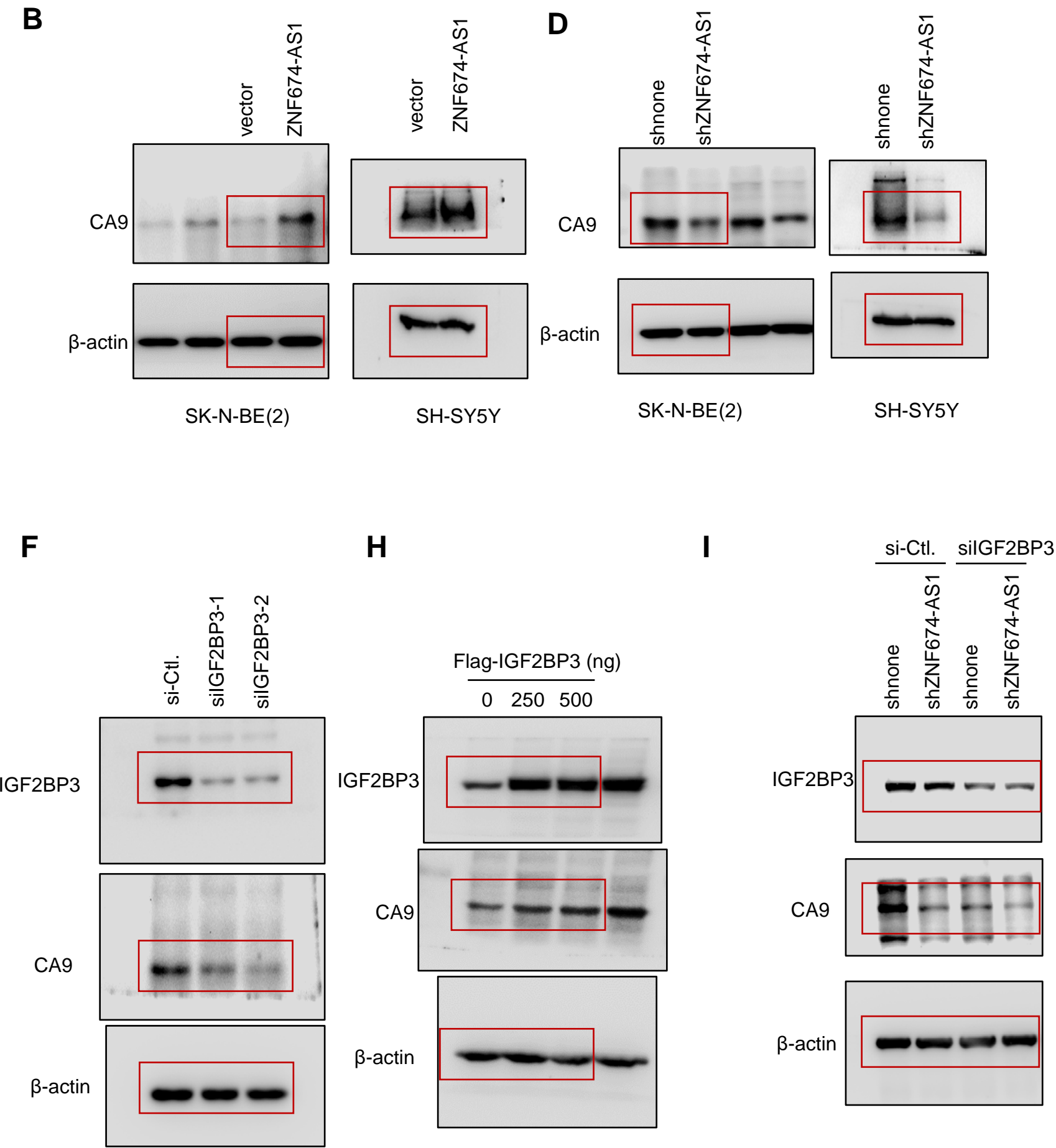

Figure S2.

F

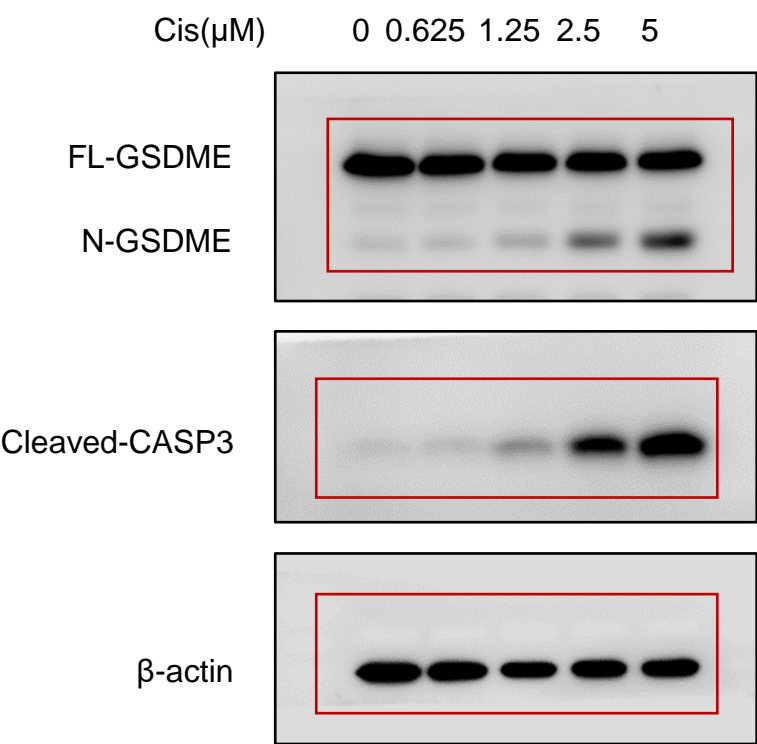

Figure S3.

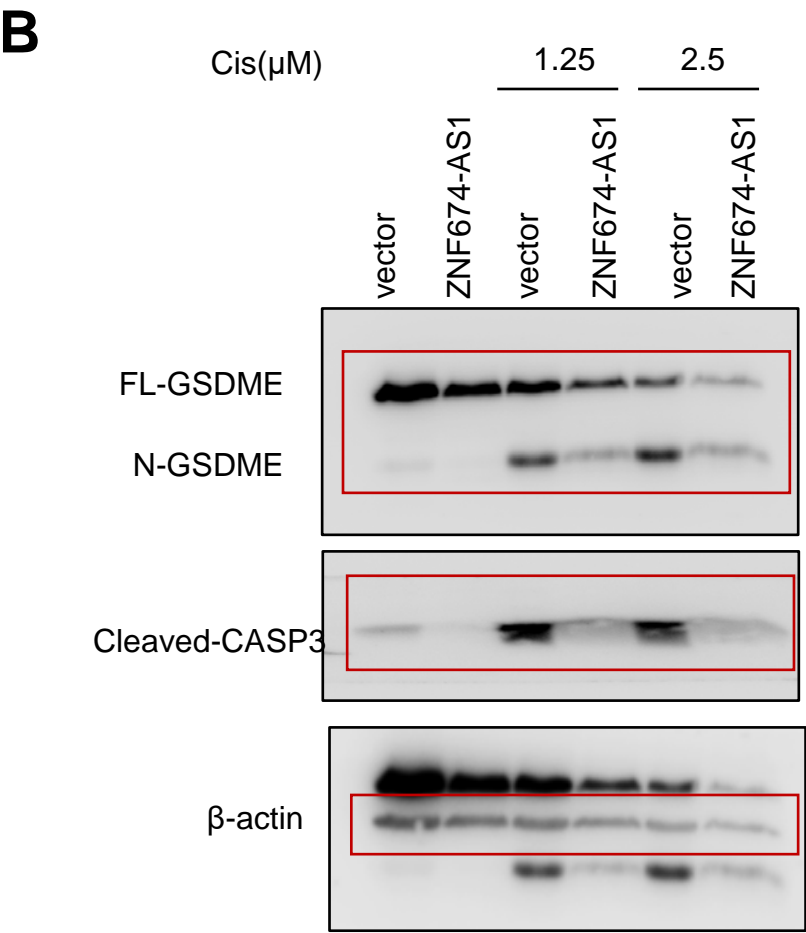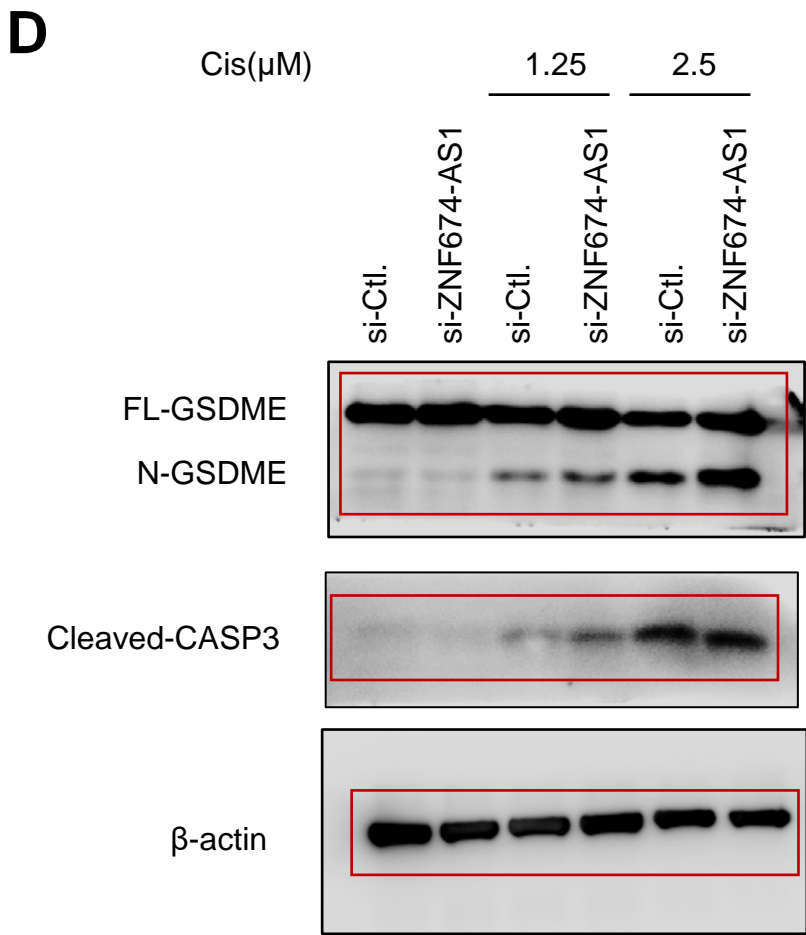

Figure S5.

A

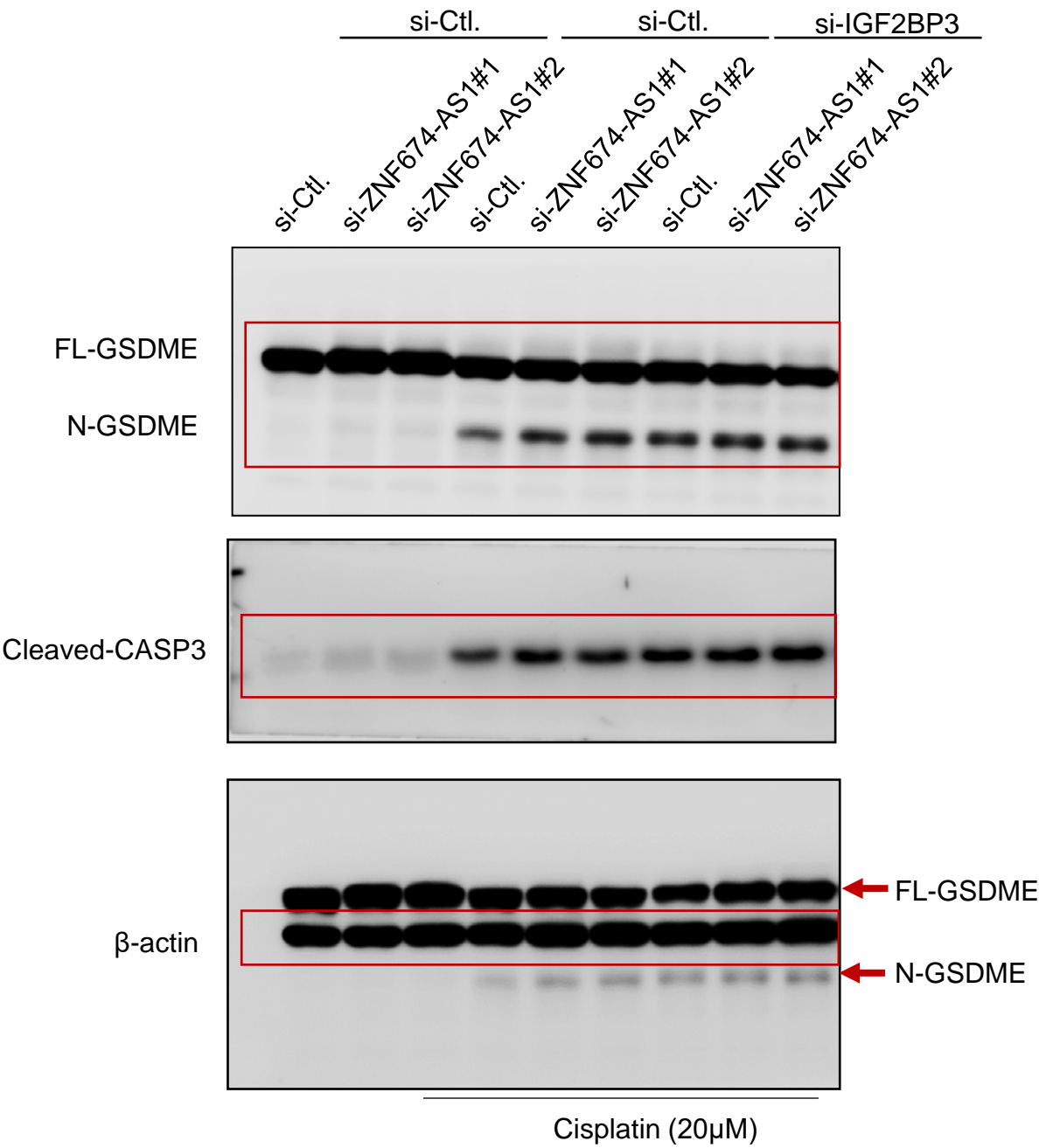

E

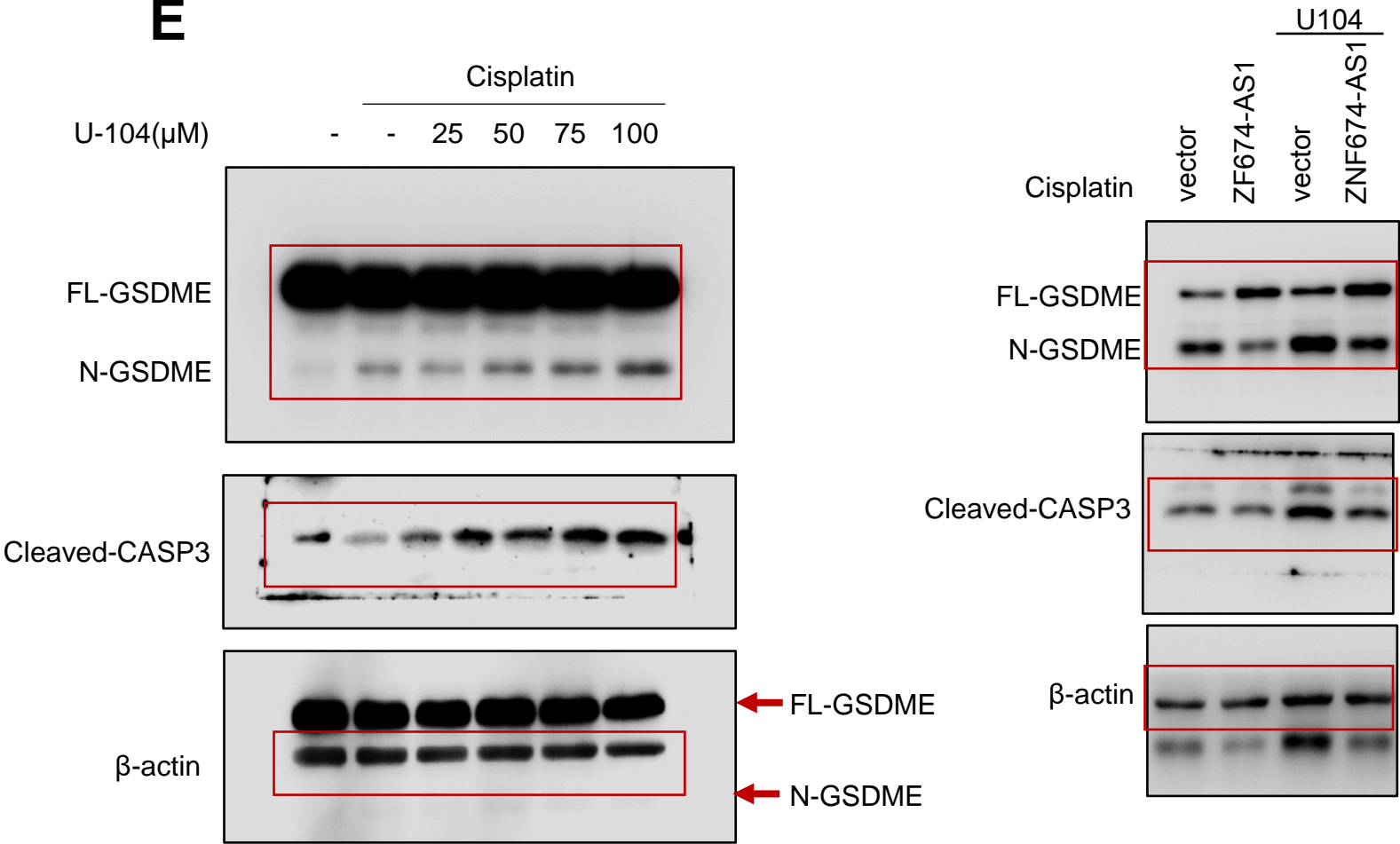

Supplement: Supplementary file 2 — Original westernblots [file 41419_2023_6394_MOESM2_ESM.pdf]
